# Cezanne promoted autophagy through PIK3C3 stabilization and PIK3C2A transcription in lung adenocarcinoma

**DOI:** 10.1038/s41420-023-01599-4

**Published:** 2023-08-18

**Authors:** Yadong Wang, Jiahao Li, Haotian Zheng, Kai Wang, Xiaoyang Ren, Guanghui Wang, Jiajun Du

**Affiliations:** 1 Institute of Oncology, Shandong Provincial Hospital, Shandong University, Jinan, People’s Republic of China; 2 Department of Thoracic Surgery, Shandong Provincial Hospital, Shandong University, Jinan, People’s Republic of China

**Keywords:** Non-small-cell lung cancer, Translational research

## Abstract

Osimertinib is a promising approved third-generation epidermal growth factor receptor tyrosine kinase inhibitor (EGFR-TKI) for treating patients with lung adenocarcinoma (LUAD) harboring EGFR-activating mutations, however, almost all patients develop resistance to Osimertinib eventually limiting the long-term efficacy. Autophagy is a vital cellular recycling process promoting Osimertinib resistance. Identifying accurate and efficient autophagy-regulatory factors is of great significance in reducing Osimertinib resistance. This study identified Cezanne, a member of the ovarian tumor protease (OTU)-deubiquitinating family, as an autophagy regulator. Cezanne was highly expressed in Osimertinib-resistant cells, and Cezanne overexpression promoted Osimertinib resistance, while chloroquine (CQ), an autophagy inhibitor, reverted this process. In the Cezanne-overexpressing cells, autophagy was activated even in the absence of autophagy inducers rapamycin and Earle’s Balanced Salt Solution (EBSS). Further study showed that Cezanne stabilized PIK3C3 by deubiquitinating K48-linked ubiquitination at Lysine 322. Surprisingly, as a compensatory mechanism of PI3P generation, PIK3C2A was shown to be upregulated by Cezanne by promoting its transcription in a POLR2A-dependent way. Based on these results, Cezanne also accelerates EGFR recycling which may explain the mechanism mediating Cezanne expression and Osimertinib resistance. In conclusion, this study establishes a new model connecting Cezanne, autophagy, and Osimertinib resistance, opening new avenues to explore the effect of Cezanne and autophagy in LUAD.

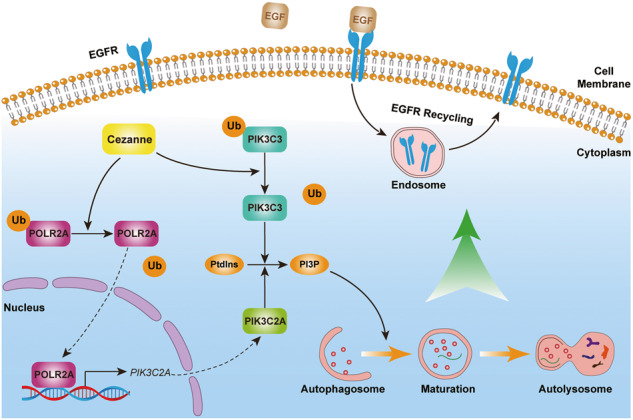

## Highlights

- Cezanne contributed to Osimertinib resistance in LUAD.
- Cezanne stabilized PIK3C3 by deubiquitinating K48-linked ubiquitination on lys322 residue.
- Cezanne promoted PIK3C2A transcription in a POLR2A-dependent way.
- Cezanne activated autophagy to accelerate EGFR recycling.

## Introduction

Lung adenocarcinoma (LUAD) is the most common pathological type of non-small cell lung cancer (NSCLC), and the incidence rate increases year by year [1]. Traditional chemotherapy has limited effects on LUAD. Epidermal Growth Factor Receptor Tyrosine Kinase Inhibitor (EGFR-TKI) has become a front-line treatment plan for patients with advanced LUAD. Although EGFR-TKI has low toxicity and high efficiency, most patients will develop drug resistance. As the third-generation EGFR-TKI drug, Osimertinib has outstanding curative and low toxic side effects. However, drug resistance may still arise [2]. Recent studies have shown that when TKI suppresses the signal pathways related to the survival of tumor cells, bypass signal activation can allow cells to retain key downstream cancer effects [2, 3]. Mechanisms of Osimertinib resistance are not fully understood [2].

Autophagy is a highly conserved biological process in that cells recycle their intracellular content to maintain cell homeostasis [4]. Autophagy activation is a complex multi-step process, mainly regulated by autophagy-related genes (ATGs) [5]. Phosphatidylinositol 3-kinase catalytic subunit type 3 (PIK3C3, also called vacuolar protein-sorting 34, VPS34) is essential for autophagosome formation, and forms functional complexes with other molecules, like Beclin-1, providing a platform for DFCP1 and WIPI2 to play an essential role in tumor development [6].

Cell autophagy activation is a drug-resistance mechanism for multiple tumor cells [6]. Autophagy helps tumor cells survive under acute stress or extreme conditions, such as extreme energy shortage, chemotherapy, and drug-caused cellular injury [6]. On the one hand, tumor cells acquire energy and material for proliferation through autophagy. On the other hand, organelles are cleared by autophagy during chemotherapy, and cells maintain intracellular stability developing resistance to targeted drugs and chemotherapy [7].

Activation of tumor cell autophagy by EGFR-TKI is a potential key mechanism for obtaining drug resistance [8]. EGFR-TKIs can induce protective autophagy in various tumor cells, such as LUAD, leading to drug resistance [9]. Osimertinib inhibits the EGFR pathway and also induces tumor cells to produce Reactive oxygen species (ROS), promoting the protective autophagy of the LUAD cells, thereby activating the bypass pathways [10]. The joint use of EGFR-TKIS and autophagy inhibitors in lung cancer cell lines show good application prospects [6, 11]. Although using EGFR-TKIs combined with lysosome inhibitors has achieved a certain clinical efficacy in clinical trials, there are still limitations in the specificity and toxicity of the drugs [6]. Exploring more accurate and efficient autophagy-regulatory factors to solve the drug resistance of third-generation EGFR-TKIs is of great significance.

Cellular zinc finger anti-NF-kappa-B protein (Cezanne, also called OTU domain-containing protein 7B, OTUD7B) is a member of the ovarian tumor protease (OTU)-deubiquitinating family. It has 1 catalytic domain (OTU) and 2 Ubiquitin-binding domains (UBD), the Ubiquitin-associated domain (UBA), and the zinc finger domain (ZnF). Cezanne is a deubiquitinating enzyme with strong activity and specificity for Lysines-11/48/63-linked ubiquitination [12–14]. Studies have shown that Cezanne influences HIF-1α transcription activity through deubiquitinating effects [15]. Cezanne also promotes the proliferation of LUAD cells by stabilizing IGF-1R [16]. Cezanne is becoming a hotspot of LUAD research and a potential target for pharmacological intervention.

This study identified Cezanne as an important factor inducing autophagy-dependent Osimertinib resistance in LUAD cells. Mechanically, Cezanne contributed to autophagy by stabilizing PIK3C3 through deubiquitinating K48-linked ubiquitination and promoting PIK3C2A transcription in a POLR2A-dependent way. This study shows a new model connecting Cezanne, autophagy, and Osimertinib resistance, which may be a potential approach to treating patients with Osimertinib resistance.

## Results

### Cezanne contributed to Osimertinib resistance in LUAD

**Fig. 1:**
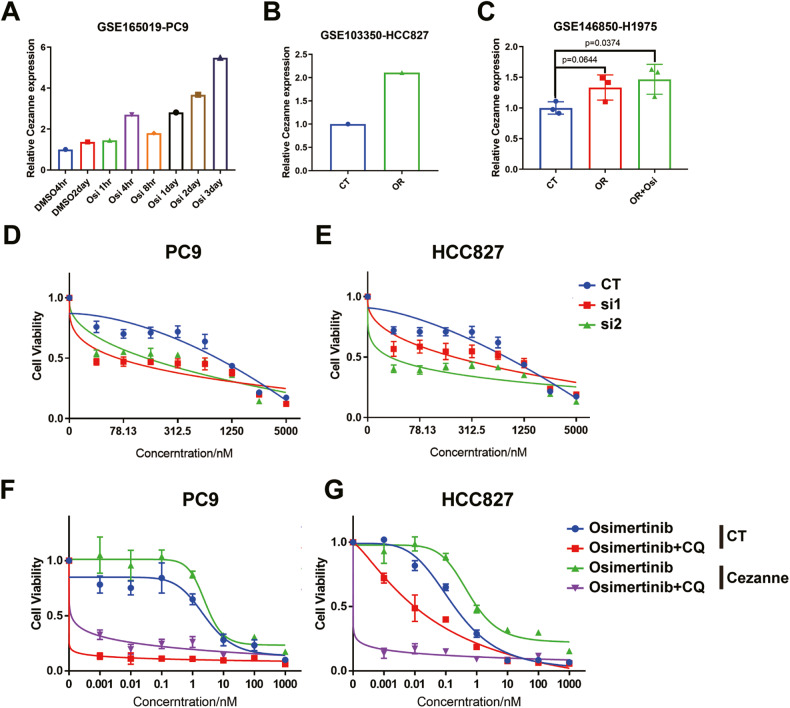
Cezanne contributed to Osimertinib resistance in LUAD. **A** Data from the GEO database (GSE165019) showed the expression change of Cezanne in PC9 cells when stimulated with Osimertinib at different times. **B** Data from the GEO database (GSE103350) showed the expression of Cezanne in HCC827-sensitive and HCC827-OR cells. OR, Osimertinib resistant. **C** data from the GEO database (GSE146850) showed the expression of Cezanne in H1975-sensitive, OR, and Osimertinib-stimulated OR cells (mean ± SD, *n* = 3.). OR, Osimertinib resistant. **D**, **E** IC50 of Osimertinib in Cezanne-OE PC9 (**D**) and HCC827 (**E**) cells decreased in Cezanne knockdown cells (mean ± SD, *n* = 6.). **F**, **G** IC50 of Osimertinib in Cezanne-OE PC9 (**F**) and HCC827 (**G**) cells was higher and the addition of CQ (5 µM) would reverse this (mean ± SD, *n* = 3.).

To investigate the connection between Cezanne and Osimertinib resistance in LUAD, analysis of the expression of Cezanne using the GEO data was conducted, which showed that Cezanne expression elevated in the Osimertinib-treated PC9 cells (Fig. 1A) and Osimertinib-resistant HCC827, H1975 cells (Fig. 1B, C). These indicated that Cezanne might promote Osimertinib resistance in LUAD cells. To test this hypothesis, we treated PC9 cells with 100 nM Osimertinib for 12 h and analyzed the change of *Cezanne* expression. Our results proved that Osimertinib stimulation would induce a higher expression of *Cezanne* (Supplementary Fig. 1A). Then we analyzed the relationship between Osimertinib sensitivity with Cezanne expression. As shown in Fig. 1D and E, Cezanne knockdown with siRNA decreased Osimertinib sensitivity in PC9 and HCC827 cells, while Cezanne-Overexpressing (Cezanne OE) cells developed Osimertinib resistance (Fig. 1F, G). Therefore, our results suggested that Cezanne contributed to Osimertinib resistance in LUAD cells.

Autophagy activation is related to Osimertinib resistance in several types of tumors [6], and our study indicated that the autophagy inhibitor CQ could inhibit the Osimertinib resistance induced by Cezanne (Fig. 1F, G), which suggested Cezanne promoted Osimertinib resistance in an autophagy-dependent way. However, the effects of Cezanne on cell autophagy were still elusive.

### Cezanne contributed to autophagy in LUAD cells

**Fig. 2:**
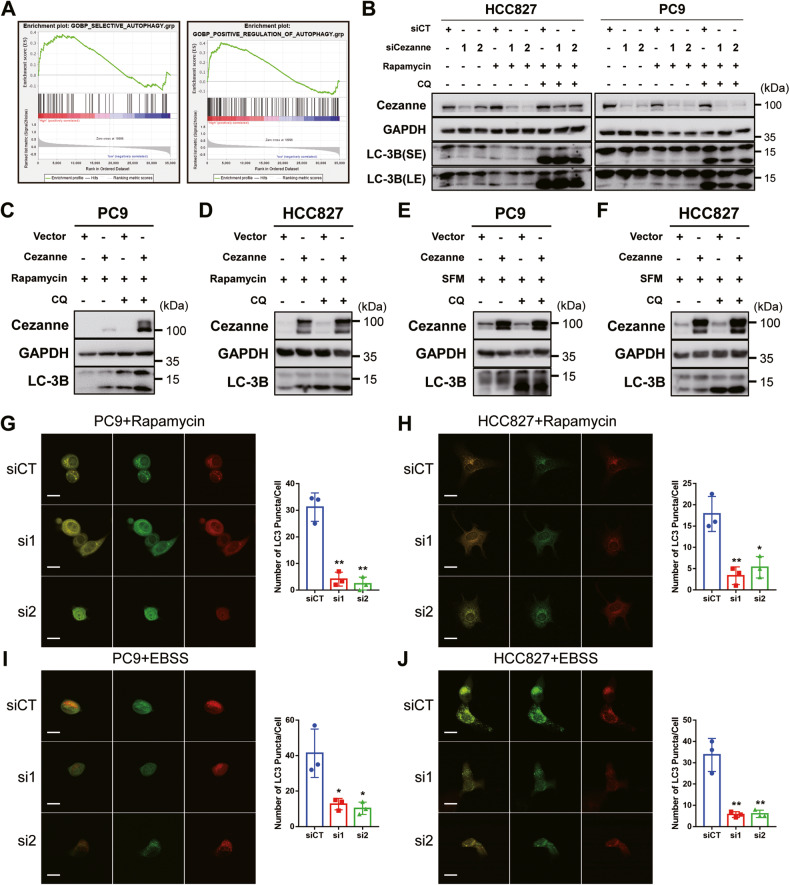
Cezanne promoted the activation of autophagy in LUAD. **A** GSEA analysis suggested the correlation between Cezanne and autophagy. **B** knockdown of Cezanne inhibited the activation of autophagy in PC9 and HCC827 cells with or without Rapamycin (5 µM) stimulation for 4 h. SE, short exposure. LE, long exposure. **C**, **D** Cezanne overexpression induced a higher level of autophagy in PC9 (**C**) and HCC827 (**D**) cells with the stimulation of Rapamycin (5 µM) for 4 h. **E**, **F** Cezanne overexpression induced a higher level of autophagy in PC9 (**E**) and HCC827 (**F**) cells after starved for 2 h. SFM, serum-free medium. **G**–**J** PC9 and HCC827 expressing mRFP-GFP-LC3 were transfected with Cezanne siRNA and then treated with Rapamycin or EBSS. The number of LC3B puncta decreased in Cezanne-knockdown cells after being stimulated with 5 μM Rapamycin (**G**, **H**) for 4 h or EBSS (**I**, **J**) for 2 h. Mean ± SD, *n* = 3. *p < 0.05, **p < 0.01, vs siCT. Scale bars: 20 µm.

Several ubiquitination-related enzymes were reported to regulate autophagy by promoting the ubiquitination or deubiquitination of some autophagy regulators, like Beclin-1 [17] and ULK1 [18]. The GSEA analysis based on TCGA data suggested the relationship between Cezanne and autophagy (Fig. 2A).

To investigate the effect of Cezanne on autophagy, we treated LUAD cell lines, PC9 and HCC827, Cezanne knockdown or overexpression, with Rapamycin or serum deprivation stimulation (Serum-free medium, SFM) to detect the autophagy flux. As shown in Fig. 2B, the knockdown of Cezanne inhibited the transformation of LC3B-I to LC-3B-II even without an autophagy inducer, and Cezanne overexpression promoted this transformation (Fig. 2C–F). Besides, CQ, an inhibitor blocking the fusion of the autophagosome with the lysosome, promoted the accumulation of LC3B-II while did not block the transformation of LC3B-I to LC3B-II (Fig. 2B–F). These results indicated that Cezanne might promote autophagy at the autophagosome formation stage.

The mRFP-GFP-LC3 was used to show the number of autophagosomes and the autophagosome maturation. So, we constructed mRFP-GFP-LC3 expressing PC9 and HCC827 cells and knock down Cezanne with siRNA to examine the number of LC3 puncta. In Fig. 2G–J, after being stimulated with 5 μM Rapamycin for 4 h or EBSS for 2 h, the number of LC3 puncta decreased significantly in Cezanne knockdown cells, which further validated the positive effect of Cezanne on autophagy activation.

### Cezanne promoted the stabilization of PIK3C3

**Fig. 3:**
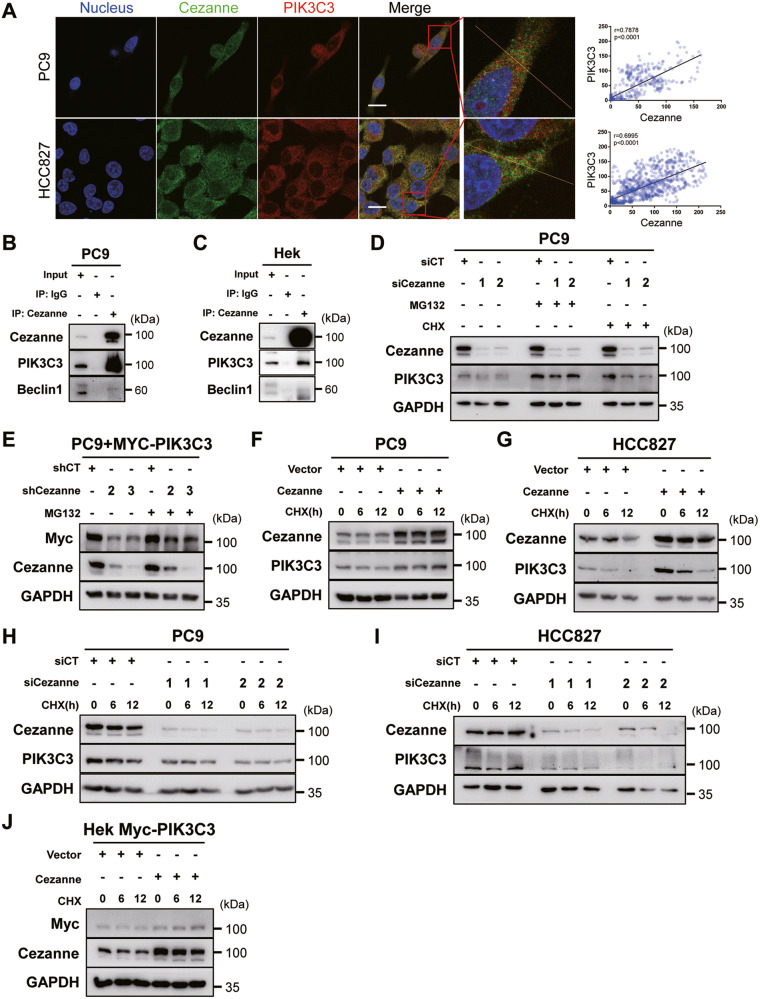
Cezanne stabilized PIK3C3. **A** Colocalization between Cezanne and PIK3C3 was examined by Confocal microscopy. The nucleus was visualized with Hoechst 33258 (blue). Intensity profiles of Cezanne and PIK3C3 were analyzed along the plotted lines by ImageJ. Pearson’s correlation coefficient in PC9 and HCC827 cells is 0.7878 (*p* < 0.0001) and 0.6995 (*p* < 0.0001), respectively. Scale bars: 20 µm. **B**, **C** CO-IP assay showed the colocalization of Cezanne and PIK3C3 in PC9 (**B**) and Hek 293T (**C**) cells. **D** Expression of PIK3C3 decreased in Cezanne knockdown cells, the addition of MG132 (20 μM) rescued the expression of PIK3C3. **E** Exogenously expressed Myc-PIK3C3 also decreased in Cezanne knockdown PC9 cells and MG132 (20 μM) would rescue the expression of Myc-PIK3C3. **F**, **G** The CHX degradation assay (CHX:100 μg/ml) indicated that Cezanne overexpression slowed down the degradation of PIK3C3 in PC9 (**F**) and HCC827 (**G**) cells. **H**, **I** the CHX degradation assay (CHX:100 μg/ml) indicated that PIK3C3 degraded faster in PC9 (**H**) and HCC827 (**I**) Cezanne knockdown cells. **J** Cezanne slowed down the degradation of PIK3C3 in Hek 293T cells.

To further elucidate the mechanism of autophagy induction by Cezanne, we tried to analyze the relationship between Cezanne and the key autophagy regulator, PIK3C3, which, connecting with Beclin-1, and the PIK3C3 regulators, PIK3R4, UVRAG, Rubicon, control the formation and maturation of autophagosome in the autophagy activation process [19]. Then, interaction analysis of Cezanne with PIK3C3 was performed using confocal microscopy, which showed the colocalization of Cezanne and PIK3C3 (Fig. 3A and Supplementary Fig. 1B). CO-IP assay showed that Cezanne could bind PIK3C3 rather than Beclin-1 (Fig. 3B, C).

Besides, we noticed that, in PC9 cells, Cezanne knockdown decreased PIK3C3 expression with or without the effect of CHX at protein level (Fig. 3D) but not mRNA levels (Supplementary Fig. 1C), and the addition of MG132 could recuse it (Fig. 3D). To further validate this effect, we constructed PC9-knockdown cell with shRNA. Then we transfected exogenous Myc-tagged PIK3C3 in shCezanne PC9 cells and found a decreased expression Myc tag in shCezanne cells and also, MG132 rescued the expression of Myc-tagged PIK3C3 (Fig. 3E), which indicated Cezanne regulated PIK3C3 at the protein level, but not at the transcription level.

To validate if Cezanne regulates the ubiquitin-proteasome degradation of PIK3C3, we conducted a CHX degradation assay. After treating PC9 and HCC827 cells with 100 µg/ml CHX to block protein synthesis for 0/6/12 h, the protein degradation was shown using western blot. The results showed that the degradation of PIK3C3 accelerated in Cezanne-knockdown cells and slowed down in Cezanne-OE cells (Fig. 3F–I). This result could be repeated in Hek 293T cells transfected with exogenously expressed PIK3C3 (Fig. 3J and Supplementary Fig. 1D). These results suggested that Cezanne could stabilize PIK3C3 by blocking the ubiquitin-proteasome degradation of PIK3C3.

### Cezanne stabilized PIK3C3 by decreasing K48-linked ubiquitination on the K322 residue

**Fig. 4:**
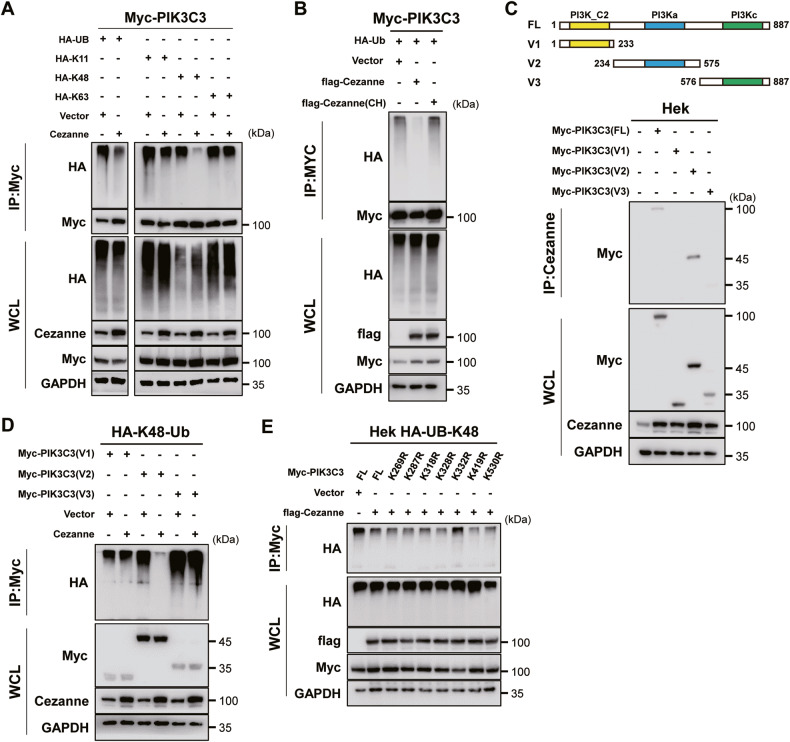
Cezanne deubiquitinates PIK3C3 at K322. **A** Immunoblot analysis of lysates from Hek 293T cells transfected with Cezanne, Myc-tagged PIK3C3 (Myc-PIK3C3) and HA-tagged ubiquitin (HA-Ub) or HA- K11/48/63-Ub followed by CO-IP with anti-Myc showed Cezanne would deubiquitinate the K48-linked ubiquitin on PIK3C3. **B** Immunoblot analysis of lysates from Hek 293T cells transfected with flag-tagged Cezanne(flag-Cezanne) or flag-Cezanne CH and Myc-PIK3C3, HA-Ub followed by CO-IP with anti-Myc showed mutation of Cezanne could not deubiquitinate PIK3C3. **C** Immunoblot analysis of lysates from Hek 293T cells transfected with Myc-PIK3C3 or Myc-PIK3C3 fragment followed by CO-IP with anti-Cezanne showed the V2 fragment was shown to be the only motif interacting with Cezanne. **D** Immunoblot analysis of lysates from Hek 293T cells transfected with Cezanne, Myc-PIK3C3 fragment, and HA-K48-Ub followed by CO-IP with anti-Myc indicated that Cezanne would deubiquitinate the K48-linked ubiquitin of V2 fragment of PIK3C3. **E** Immunoblot analysis of lysates from Hek 293T cells transfected with Cezanne, Myc-PIK3C3, or lysine-mutation PIK3C3 and HA-K48-Ub followed by CO-IP with anti-Myc to analyze the ubiquitination of constructed lysine-mutation PIK3C3.

As an OTU-deubiquitination enzyme, Cezanne targets K11, K48, and K63-linked ubiquitination [12–14]. To define the type of linkages in the polyubiquitin chains attached to PIK3C3, we constructed HA-tagged ubiquitin and lysine-mutated ubiquitin, that all the lysine residues were replaced by arginine except for the only one at K11, K48 or K63. We then transfected Hek 293 T cells with HA-tagged ubiquitin (HA-Ub) and K11/48/63-Ub. CO-IP assays showed that Cezanne OE decreased PIK3C3 ubiquitination and induced a significant decrease of the K48-linked ubiquitination and a slight decrease of K11-linked ubiquitination of PIK3C3 (Fig. 4A), which were reported to mediate the degradation of target molecules [20]. Besides, the enzymatic activity mutant Cezanne (C194S and H358R, Cezanne CH) could not decrease PIK3C3 ubiquitination (Fig. 4B), further confirming the deubiquitination effect of Cezanne on PIK3C3.

To explore the motif or molecule basis of Cezanne and PIK3C3, we constructed plasmids composed of different part of these two proteins shown in Fig. 4C and Supplementary Fig. 1E. The CO-IP assay showed that the V2, containing PI3Ka domain, of PIK3C3 was the Cezanne binding region, and C3, containing C-terminal ZnF domain, was the PIK3C3 binding part on Cezanne (Fig. 4C and Supplementary Fig. 1E). Furthermore, immunoblot analysis of lysates from Hek 293 T cells transfected with K48-Ub, Myc-tagged PIK3C3 fragments showed the ubiquitination of V2 fragment of PIK3C3 was inhibited by Cezanne which indicated that the actual K48-linked ubiquitination lysine residue of PIK3C3 might locate at this fragment (Fig. 4D).

According to the conserved lysine residues of PIK3C3 from NCBI (Supplementary Fig. 2), we mutated 7 lysine sites to arginine (K to R) to validate the accurate ubiquitinated lysine residue on PIK3C3. Figure 4E showed that only K322R of PIK3C3 lost the deubiquitination effect of Cezanne, indicating that K48-linked ubiquitination modulated by Cezanne on PIK3C3 located on K322 residue.

### Cezanne controls the transcription of PIK3C2A in a POLR2A-dependent way

**Fig. 5:**
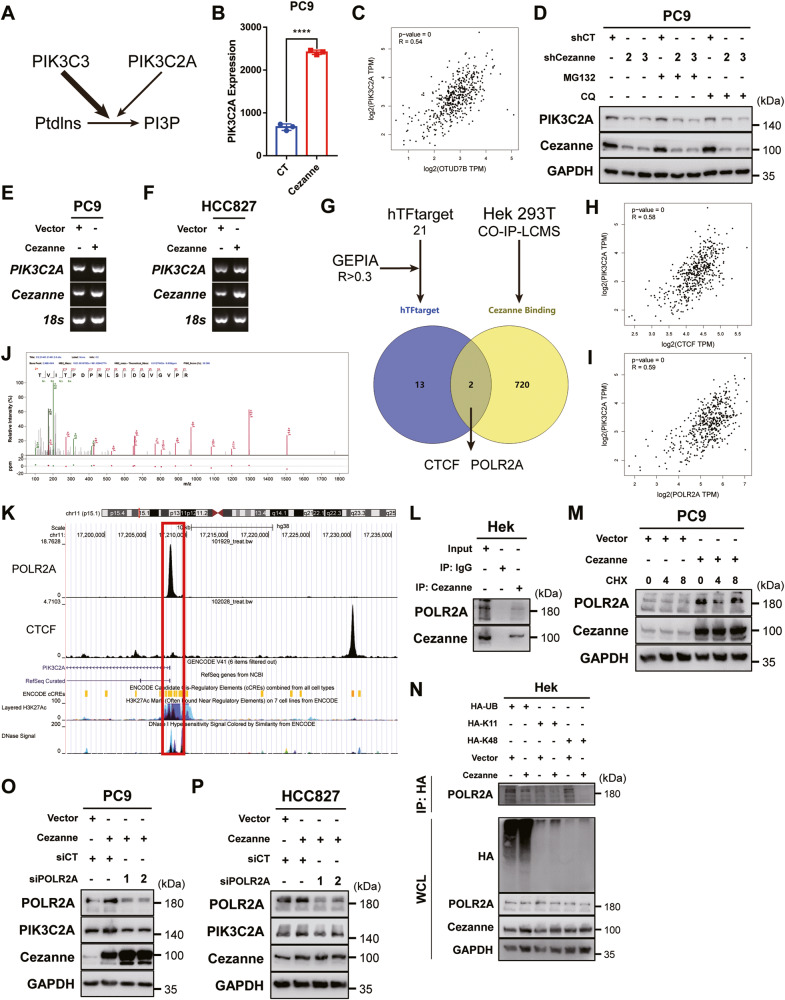
Cezanne promotes the transcription of PIK3C2A in a POLR2A-dependent way. **A** The effect of PIK3C3 and PIK3C2A on PI3P production. **B** PIK3C2A is highly expressed in Cezanne OE PC9 cells. Mean ± SD, *n* = 3. ****P < 0.0001, vs. CT. **C** the co-expression analysis of Cezanne and PIK3C2A in the GEPIA database. **D** MG132 (20 μM) and CQ (40 µM) fail to rescue the decreased expression of PIK3C2A in Cezanne knockdown cells. **E**, **F** the mRNA of PIK3C2A elevated in Cezanne OE PC9 and HCC827 cells. **G** The screen of possible transcription factor of PIK3C2A using hTFtarget database, GEPIA, and LCMS data. **H**, **I** co-expression analysis of CTCF (**H**), POLR2A (**I**) with PIK3C2A in the GEPIA database. **J** the LCMS/MS data showed the peptide of POLR2A acquired from immunoprecipitated with Cezanne antibody. **K** CHIP-seq data from Cistrome Project shown in the UCSC browser, that only POLR2A could bind to the transcription start site of PIK3C2A. **L** CO-IP assay showed the interaction between Cezanne and POLR2A. **M** CHX degradation assay showed that Cezanne stabilized POLR2A. **N** CO-IP assay immunoprecipitated with anti-HA antibody indicated that Cezanne contributed to the deubiquitination of K48-linked ubiquitin. **O**, **P** knockdown of POLR2A induced a decreased expression in Cezanne OE PC9 and HCC827 cells.

Synthesis of PI3P was the main mechanism by PIK3C3 to regulate autophagy, and a recent study reported that PIK3C2A was an alternative way to promote PI3P production (Fig. 5A) [19, 21]. So, we explored the effect of Cezanne on *PIK3C2A* and found that in the sequencing data, *PIK3C2A* expression elevated in Cezanne OE cells (Fig. 5B), and data from GEPIA database (http://gepia.cancer-pku.cn/) showed that *Cezanne* expression was positively related to that of *PIK3C2A* (Fig. 5C). Neither the proteosome inhibitor, MG132 nor the autophagy inhibitor, CQ could rescue the downregulation of PIK3C2A in PC9 shCezanne cells (Fig. 5D). PCR results confirmed the elevation of *PIK3C2A* in Cezanne OE cells (Fig. 5E, F). All these results suggested that Cezanne might promote PIK3C2A transcription, but not protein stability.

As we did not find any report that Cezanne could serve as a transcriptional factor (TF) or co-activator, we tried to identify the mediator between Cezanne and PIK3C2A. First, CO-IP assay in Hek 293T cells, IP with anti-Cezanne, was conducted and the extract was sent for LC-MS/MS analysis. Then, Eighteen TFs were predicted through the hTFtarget database (http://bioinfo.life.hust.edu.cn/hTFtarget#!/) to regulate PIK3C2A expression in the lungs. And finally, we compared the correlation between these TFs and PIK3C2A in the GEPIA database. TFs with *R* > 0.3 (Pearson coefficient, GEPIA) were selected to interact with proteins identified by CO-IP-LCMS (Fig. 5G). Only two TFs were selected to be the candidate, POLR2A and CTCF (Fig. 5H–J). However, the public CHIP data from the Cistrome project (http://cistrome.org/) showed that POLR2A, rather than CTCF, binds the transcription start site of PIK3C2A (Fig. 5K) and we chose POLR2A as the candidate TF.

CO-IP assay in Hek 293T cells, IP with anti-Cezanne, validated the binding of Cezanne and POLR2A (Fig. 5L). The following CHX degradation assay showed that expression of POLR2A was positively correlated to that of Cezanne at the protein level because degradation of POLR2A slowed down in Cezanne OE cells (Fig. 5M). Figure 5N showed that Cezanne promoted deubiquitination of K48-linked ubiquitin of POLR2A, and POLR2A knockdown would decrease the PIK3C2A expression in Cezanne OE cells (Fig. 5O, P). All these indicated that Cezanne could induce PIK3C2A expression by stabilizing PLOR2A.

### PIK3C3 and PIK3C2A are the main mediators between Cezanne and Autophagy

**Fig. 6:**
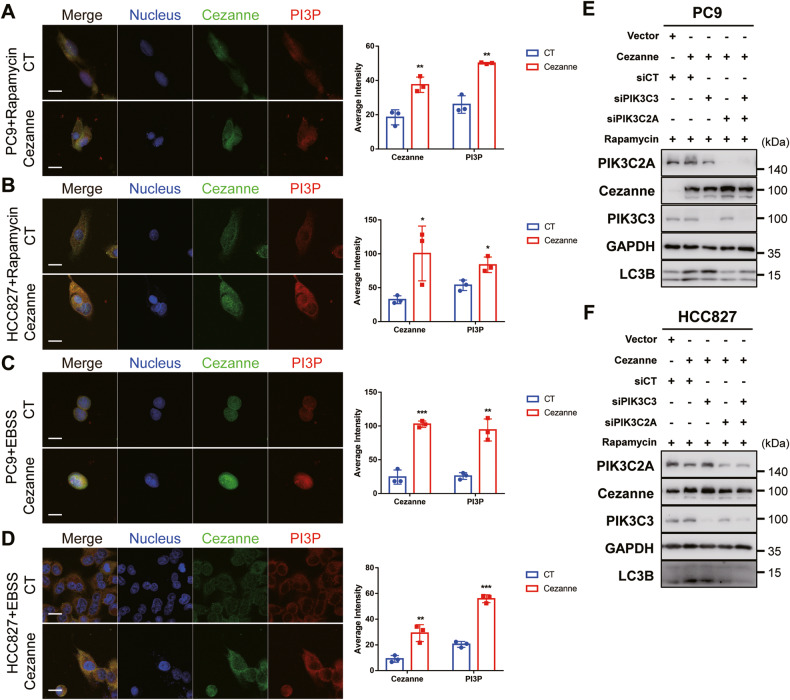
Cezanne promoted autophagy through PIK3C3 and PIK3C2A. **A**–**D** IF assay to compare the PI3P production. The fluorescence intensity of GST-2×FYVE labeled PI3P increased in Cezanne OE cells. Quantification of the average intensity of indicated proteins was analyzed by ImageJ. Mean ± SD, *n* = 3. *p < 0.05, **p < 0.01, ***p < 0.001, vs. CT. Scale bars: 20 µm. **E**, **F** knockdown of PIK3C3 and PIK3C2A inhibited Cezanne-induced autophagy in cells treated with 5 µM Rapamycin for 4 h.

PIK3C3 and PIK3C2A promote autophagy through catalyzing PtdIns to PI3P, which helps autophagosome membrane extension and maturation [19]. Hereafter, we detected the level of PI3P in cells after being treated with Rapamycin or EBSS. FYVE is a peptide that specifically binds to PI3P in cells which have been widely used [21–23]. Then we constructed GST-2×FYVE plasmid and the purified GST-2×FYVE was validated (Supplementary Fig. 1F). After incubating PC9 and HCC827 cells with purified GST-2×FYVE, confocal microscopy was used to record the fluorescence and then the fluorescence intensity was calculated by ImageJ. As shown in Fig. 6A–D, the GST-tag fluorescence intensity is greater and the quantity is more in Cezanne OE cells, suggesting more production of PI3P in Cezanne OE cells.

To further illustrate the effect of PIK3C3 and PIK3C2A on autophagy activation promoted by Cezanne, we used siRNA to knock down PIK3C3 and PIK3C2A. The results showed that under Rapamycin stimulation, PIK3C3 or PIK3C2A knockdown reversed the autophagy activation induced by Cezanne, and the knockdown of both would be more remarkable (Fig. 6E, F).

### Cezanne promoted Osimertinib resistance by accelerating EGFR recycling

A recent study proved that autophagy would accelerate EGFR recycling under the stimulation of EGF [9], contributing to Osimertinib resistance. Cezanne was shown to activate cell autophagy, making us wonder whether Cezanne could promote Osimertinib resistance in an autophagy-dependent way.

**Fig. 7:**
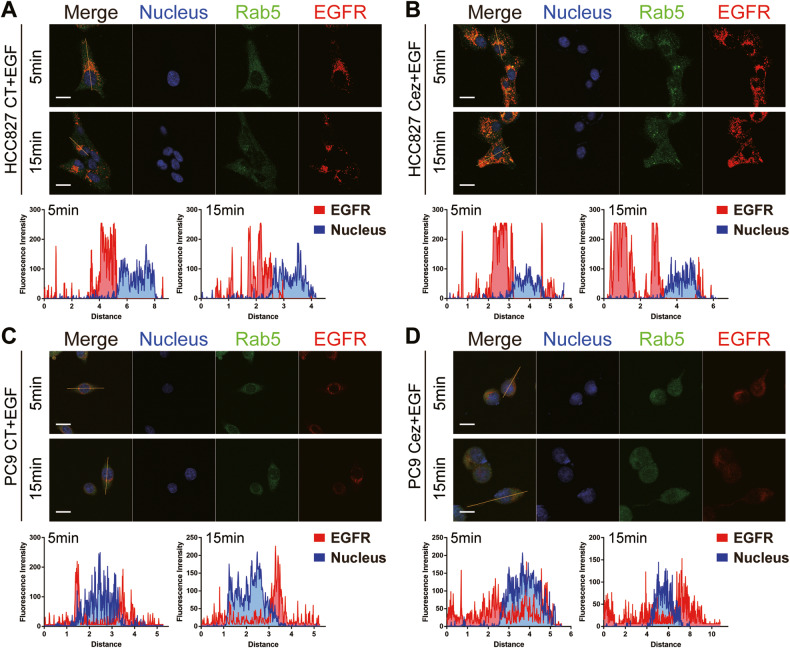
Cezanne promoted EGFR recycling. Cezanne accelerates EGFR endocytic trafficking after being stimulated with 50 ng/ml EGF in HCC827 (**A**, **B**) and PC9 (**C**, **D**). Intensity profiles of indicated proteins along the plotted lines were analyzed by ImageJ to show the distribution of EGFR in cells after being stimulated with 50 ng/ml EGF for 5 or 15 min. The nucleus was visualized with Hoechst 33258 (blue). Confocal imaging results are representative of three independent experiments. Cez, Cezanne. Scale bars: 20 µm.

To validate this hypothesis, we treated PC9 and HCC827 cells with 50 ng/ml EGF and captured the distribution of EGFR and an early-endosome marker, Rab5, at 5 and 15 min using confocal microscopy. The results showed that after 5 min stimulation with EGF, most EGFRs gathered around the nucleus, and only a few of the red dots in the Cezanne OE cells recycled away from nucleus (Fig. 7). However, at 15 min, the fluorescence intensity analysis along the cell long axis showed that many red dots (EGFRs) distributed far away from nucleus, indicating most EGFRs in Cezanne OE cells already recycle into the membrane (Fig. 7B, D), while most red dots in the CT group gathered around the nucleus (Fig. 7A, C). These results suggested that Cezanne could promote autophagy activation and accelerate the recycling of EGFR, which facilitated Osimertinib resistance.

## Discussion

**Fig. 8:**
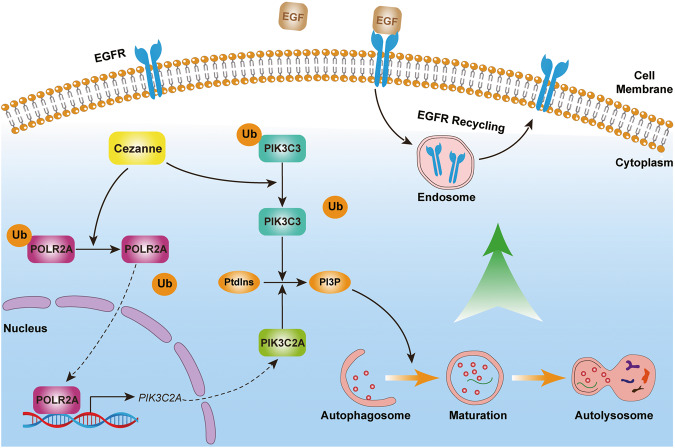
Regulation of Cezanne in autophagy and Osimertinib resistance in LUAD. Cezanne promoted autophagy through PIK3C3 stabilization and PIK3C2A transcription, which contributed to Osimertinib resistance in LUAD.

Autophagy is a degradation and recycling system, that plays a dynamic tumor-suppressive or tumor-promoting role in different contexts and stages of cancer development [24]. When tumors are subjected to environmental stresses, autophagy will contribute to the survival and growth of the tumor cells and promote the aggressiveness of the cancers [24]. In addition to its role in tumor progression, autophagy promotes drug resistance in tumor cells [25, 26]. In this study, we started with the Osimertinib resistance-related gene expression analysis in LUAD cells. From the public GEO data, we found a positive relationship between Cezanne expression and Osimertinib resistance or treatment (Fig. 1A–C). The IC50 assay also proved the resistance of Osimertinib induced by Cezanne and the addition of CQ significantly decreased the Osimertinib resistance in LUAD cell lines, PC9 and HCC827 cells (Fig. 1F, G), which suggested Cezanne promoted Osimertinib resistance and autophagy played a vital role (Fig. 8).

Then, to validate the hypothesis, we conducted a further study to investigate the autophagy flux change which showed that Cezanne could promote autophagy activation, that Cezanne promoted the transformation of LC3B-II and the formation of autophagosome (Fig. 2B–F). Therefore, we constructed a model that Cezanne could promote LUAD Osimertinib resistance through autophagy (Fig. 8).

Ubiquitination and deubiquitination control the initiation, execution, and termination of autophagy [27]. Many autophagy effectors, including ULK1 [28, 29], Beclin-1 [30, 31], and ATG14 [32], were reported to be ubiquitinated or deubiquitinated and changed their stability which regulated the autophagy flux. Our study revealed that Cezanne regulated the stability of PIK3C3 (Fig. 3F–I) and a proteasome inhibitor, MG132, could inhibit the PIK3C3 degradation (Fig. 3D, E, J), which indicated the effect of Cezanne on the ubiquitin-proteasome pathway degradation of PIK3C3. As Cezanne is a deubiquitination enzyme that specifically targets K11-, K48-, and K63-linked ubiquitination, and generally, K11- and K48-linked ubiquitination chain was related to protein degradation [20], we compared these 3 kinds of ubiquitination on PIK3C3. The results indicated that Cezanne significantly decreased the level of K48-linked ubiquitination of PIK3C3 (Fig. 4A). Surprisingly, there was also a slight reduction of K11-linked ubiquitination in Cezanne OE cells which implied that Cezanne might regulate the stability of PIK3C3 through deferent ways.

The phosphatidylinositol 3-kinases (PtdIns3Ks) and the phosphoinositide 3-kinases (PI3Ks) are the most pivotal groups of kinases controlling autophagy. PtdIns3Ks and PI3Ks are grouped into 3 classes in mammalian cells based on their structural features and substrate specificities. PIK3C2A belongs to Class II PI3Ks, and PIK3C3 belongs to Class III PtdIns3K. Both Class II PI3Ks and Class III PtdIns3K could phosphorylate PtdIns to generate PI3P, the most pivotal positive regulator of autophagy [33, 34]. PI3P acts as a platform for the assembly and coordination of autophagy-regulatory proteins and also as a signaling molecule for autophagosome formation during the autophagic process [19].

Studies showed that PI3P generation and autophagic function rely predominantly on PIK3C3 activity [35, 36], while the research from Boukhalfa et al. also showed that PIK3C2A might be a compensatory way to generate PI3P [21]. We first discovered that in Cezanne OE cells, the expression of PIK3C2A also increased. The public data validated the co-expression between Cezanne and PIK3C2A (Fig. 5B, C). These results implied that Cezanne might regulate the transcription of PIK3C2A. Figure 5D showed that MG132 and CQ could not reverse the decrease of PIK3C2A in Cezanne knockdown cells, which further supported our hypothesis.

Regulation of transcription was an important way to control autophagy. The most studied transcription factor, transcription Factor EB (TFEB), is a main lysosomal and autophagic function regulator and is widely studied for regulating various ATGs [37, 38]. However, there was no report that Cezanne could act as a transcription factor or co-activator, we had to find the factor that mediates Cezanne and the regulation of PIK3C2A transcription. In this study, with the help of a public database and LC-MS/MS analysis, we identified a transcription factor, called POLR2A, which might be the mediator between Cezanne and PIK3C2A. Indeed, Cezanne could bind and stabilize POLR2A and deubiquitinate the K48-linked ubiquitination of POLR2A (Fig. 5L–N). POLR2A knockdown would reverse the effect of Cezanne on PIK3C2A (Fig. 5O, P). All these results suggested that Cezanne could act as a deubiquitination enzyme to deubiquitinate K48-linked ubiquitination and stabilize POLR2A, then promote the transcription of PIK3C2A in a POLR2A-dependent way.

In our study, Cezanne colocalized with PIK3C3 and stabilized PIK3C3 by slowing down its ubiquitin-proteasome pathway degradation (Fig. 3), and we also found Cezanne promoted PIK3C2A transcription (Fig. 5). Consistently, based on the regulation of PIK3C3 and PIK3C2A, IF assay confirmed the elevated PI3P synthesis in Cezanne OE cells (Fig. 6A–D), which further support the effect of Cezanne on autophagy.

Activated EGFR and other Receptor Tyrosine Kinases (RTKs) are internalized by clathrin-mediated endocytosis or clathrin-independent means under particular conditions [39]. Moreover, recent studies showed that stabilization or repaid recycling of RTK would promote the targeted-therapy resistance [9, 40–43]. Besides, Fraser et al. found that autophagy inhibition could reduce EGF-mediated signaling by prolonging the residence of EGFR [9]. As Cezanne could activate cell autophagy, in our study, in low- and high-Cezanne expression cells, we compared the EGFR recycling speed by focusing on the EGFR location in cells. Consistently, compared with the focused distribution of EGFR after a 5 min stimulation, Cezanne OE would induce a more scattered distribution of EGFR after a 15 min stimulation of EGF, indicating that Cezanne could accelerate EGFR recycling (Fig. 7). And this result connected Cezanne, autophagy and Osimertinib resistance.

Autophagy is a vital cellular catabolic process that results in the lysosome-mediated recycling of organelles and protein aggregates. Autophagy’s activation regulates different cellular functions or cell destiny in different tissue [24]. The numerous ATGs and steps also complicate autophagy regulation. This study proposes a new mechanism for the deubiquitinating enzyme Cezanne to activate autophagy by stabilizing PIK3C3 and promoting PIK3C2A transcription, and we identified a new pathway that Cezanne regulates gene expression. However, there are still some deficiencies. First, we did not screen a specific inhibition compound of Cezanne and we could not validate the effect of Cezanne inhibition on EGFR resistance. Second, in GSEA analysis, as a differently expressed genes (DEGs) related function analysis, we did not study the DEGs that might control autophagy and other ATGs also changed in the Cezanne OE cells in the sequencing data. Further experimentation is needed to validate the results. Third, Cezanne was reported to regulate the stability of p62 [44], so in this study, we did not show the results of p62.

In conclusion, this study showed a new network connecting Cezanne, autophagy, and Osimertinib resistance, and in consideration of the wide effect and complexity of autophagy, further study is needed to explore the effect of autophagy induced by Cezanne on other cellular functions.

## Materials and methods

### Cell culture

PC9 and HCC4006 cells were cultured in RPMI 1640 (VivaCell Biotechnology GmbH, Germany) with 10% fetal bone serum (GIBCO, USA). HCC827, H1975, and Hek 293T cells were cultured in DMEM (VivaCell). Every 1–2 days, the medium was replaced with fresh medium, and the cells were passaged at the confluence. The cells were authenticated by STR profiling and examined for mycoplasma contamination by polymerase chain reaction (PCR). Stable-transfected cells were cultured with a medium with puromycin (Invitrogen, CA, USA). For autophagy induction, cells were starved by incubating them in Earle’s Balanced Salt Solution (EBSS) medium (Beyotime, Shanghai, China) or Rapamycin(5 μM) treatment. Lysosomal degradation was inhibited by 40 µM chloroquine (CQ, MCE, Shanghai, China).

### Data sources and bioinformatics analysis

Gene Expression Omnibus (GEO) datasets (www.ncbi.nlm.nih.gov/geo; GSE165019, GSE103350, GSE146850) were downloaded to analyze the differentially expressed genes associated with Osimertinib treatment. Gene Set Enrichment Analysis (GSEA) based on the cancer genome atlas (TCGA) data (www.tcga-data.nci.nih.gov/tcga) was used to analyze the correlation between differential gene expression and cellular pathways. Gene Expression Profiling Interactive Analysis (GEPIA, http://gepia.cancer-pku.cn/) [45], an interactive web server for analyzing the RNA-sequencing expression data developed by Tang et al. was used to analyze the gene co-expression using the “Correlation Analysis” function. Cistrome Data Browser (http://cistrome.org/db/#/) was used to identify the POLR2A (No. 101929) and CTCF (No. 102028) ChIP-seq data.

### Total RNA extraction and quantitative real-time polymerase chain reaction (qRT-PCR)

Total RNA was extracted using the AG RNAex Pro Reagent (Accurate Biotechnology, Changsha, Hunan, China), and reverse transcription was performed with the Evo M-MLV RT Premix for PCR (Accurate Biotechnology). The cDNA was examined by agarose gel electrophoresis or qRT-PCR.

The cDNA was subjected to quantitative RT-PCR using the SYBR Green Premix Pro Taq HS qPCR Kit (Accurate Biotechnology). The assay was performed on a Light Cycler 480II (Roche, Switzerland) following the manufacturer’s instructions. The 18 S ribosomal RNA was used to normalize the cDNA amount between samples. Primer sequences are as follows: 18S forward 5′-AAACGGCTACCACATCCAAG-3′, reverse 5′-CCTCCAATGGATCCTCGTTA-3′. Cezanne forward 5′-ATGTCCGATTGGCCAGTGTAA-3′, reverse 5′-TCCAGACTCAGGAGTGGACC-3′. POLR2A forward 5′-AGTCCGGATGAACTGAAGCG-3′, reverse 5′-CACGTGAAACACAGGCTTGG-3′. PIK3C2A forward 5′-GCAGCTTGAGGCCTTGCTAT-3′, reverse 5′-TGGTGCTGCTTGACAACTCA-3′. PIK3C3 forward 5′-GCTGTCCTGGAAGACCCAAT-3′, reverse 5′- CTTGGCGAAACATGCCGTAT-3′.

### Western blotting

Proteins were extracted from cells with RIPA buffer complemented with phenylmethanesulfonyl fluoride (PMSF, Beyotime). Protein extracts were subjected to sodium dodecyl-sulfate polyacrylamide gel electrophoresis (SDS-PAGE) and transferred to nitrocellulose membranes. Immunoblots were blocked with 5% bovine serum albumin (BSA) in TBS/Tween 20 and incubated with primary antibodies overnight at 4 °C. The bands were visualized with the FluorChem E System (Protein Simple, USA).

The following antibodies diluted in 5% BSA were used: anti-GAPDH (sc-47724, 1:500), anti-p62 (sc-28359, 1:1000), and anti-Beclin-1 (sc-48341, 1:1000) were purchased from Santa Cruz Biotechnology. Anti-LC3B (3868 T, 1:2000) and anti-PIK3C3 (4263S, 1:2000) were purchased from Cell signal technology (CST). Anti-Cezanne (16605-1-AP, 1:1000), anti-POLR2A (20655-1-AP, 1:1000), anti-PIK3C2A (22028-1-AP, 1:1000), and anti-Flag (20543-1-AP, 1:1000) were purchased from Proteintech (Wuhan, Hubei, China). Anti-Myc (TA150121, 1:1000), anti-HA (TA180128, 1:1000), and anti-GST (TA150101, 1:1000) were purchased from Origene (Wuxi, Jiangsu, China).

### Cell viability assay

A Sulforhodamine B (SRB) Assay was conducted to detect cell proliferation in the half-maximal inhibitory concentration (IC50) detection assay. Briefly, cells were plated in a 96-well dish at a density of 2000 cells per well. After 6–12 h, when all cells were attached, we added Osimertinib and/or CQ into the plate and treated for 72 or 96 h. Cells were fixed with 60 μl of 10% cold trichloroacetic acid (TCA) per well and incubated at 4°C for at least 6 h. The plates were washed five times and kept overnight at room temperature. Staining was performed by adding 60 μl of 0.057% (wt/vol) SRB solution to each well for 20 min. The plates were washed with 1% acetic acid and kept overnight at room temperature. The plates were incubated with 150 μl of 10 mM Tris and shaken. The absorbance at 562 nm was measured in a microplate reader (Thermo Fisher, USA), and the results were analyzed with GraphPad Prism7 (GraphPad Software, USA).

### Immunoprecipitation assay

Cells were lysed in lysis buffer (Beyotime, with PMSF) on ice for 30 min. Supernatants were collected and incubated with protein G Plus-Agarose Immunoprecipitation reagent (Santa Cruz Biotechnology, sc-2003) together with primary antibody at 4 °C overnight. Then the beads were washed five times with lysis buffer, and samples were resuspended with 1% SDS and boiled at 100 °C for 5 min, followed by western blot.

### Plasmids and RNA interference assay

Wild-type (WT) OTUD7B, PIK3C3, and their fragment plasmids were obtained from Beijing Syngenbio Co., LTD. (Beijing, China). The HA-K11, -K48, -K63, and -Ub plasmids were acquired from the MiaoLing Plasmid Platform (Wuhan, Hubei, China). Lipofectamine 3000 (Invitrogen) was used for plasmid transfection according to the manufacturer’s instructions.

The siRNAs were synthesized by Beijing Syngenbio Co., LTD. (Beijing, China) as follows: siCT: 5’-UUCUCCGAACGUGUCACGUTT-3’ and 5’-ACGUGACACGUUCGGAGAATT-3’; siCezanne-1: 5’-GGAUGACAUCGUUCAAGAATT-3’ and 5’-UUCUUGAACGAUGUCAUCCTT-3’; siCezanne-2: 5’-GUUCUGAGGAGCCUGUAUATT-3’ and 5’-UAUACAGGCUCCUCAGAACTT-3’; siPIK3C3: 5’-GAGAUGUACUUGAACGUAAUGTT-3’ and 5’-CAUUACGUUCAAGUACAUCUCTI-3’; siPIK3C2A: 5’-CCACUUAUGCUUUACCUUCUATT-3’ and 5’-UAGAAGGUAAAGCAUAAGUGGTT-3’; siPOLR2A-1: 5’- CCUGGUGAAGACAAUGAAATT-3’ and 5’- UUUCAUUGUCUUCACCAGGTT-3’; siPOLR2A-2: 5’-GCGGCAGACGUUUGAGAAUTT-3’ and 5’-AUUCUCAAACGUCUGCCGCTT-3’.

According to the manufacturer’s instructions, these siRNAs were transfected into cells using jetPRIME® transfection reagent (Polyplus, Shanghai, China).

### Cycloheximide (CHX) protein degradation assay

CHX protein degradation assay was performed by incubating the cells in a medium containing 100 µg/ml CHX (MCE) at 37 °C. Cells were lysed with the lysis buffer every 4 or 6 h after CHX incubation.

### Immunofluorescence staining (IF)

The culture medium was removed, and cells were washed 3 times with 1× PBS for 10 min. PBS was added slowly along the wall of the plate to avoid washing away the cells. Then, 2 ml methanol was added to each well, and plates were incubated at room temperature for 20 min. After washing three times, cells were blocked for 1 h in 5% BSA and incubated with primary antibody overnight in 5% BSA, followed by incubation with Conjugate secondary antibody (CST). Nuclei were stained with Hoechst 33258 (Beyotime). Cells were examined by confocal laser microscopy (Leica, Germany). The fluorescence intensity was calculated by ImageJ (National Institutes of Health, USA). The following antibodies diluted in 5% BSA were used: anti-PIK3C3 (sc-365404, 1:200), anti-EGFR (sc-373746, 1:200) from Santa Cruz Biotechnology, Anti-Rab5 (3547T, 1:1000) from CST. Anti-Cezanne (16605-1-AP, 1:1000) from Proteintech, anti-GST (TA150101, 1:1000) from Origene.

### mRFP-GFP-LC3

Tumor cells were transfected with mRFP-GFP-tagged LC3 adenovirus (Genechem, Shanghai, China) for 24 h. Then we knockdown Cezanne with siRNA. Images were acquired using a confocal microscope (Leica) with a ×100 oil objective. Autophagosomes were detected as yellow dots (RFP + GFP + ), while mature autolysosomal organelles were detected as red-only dots.

### Protein purification

Recombinant FYVE with a GST-tag was expressed in BL21 *E. coli* induced with 500 µM isopropyl β-d-1-thiogalactopy-ranoside (IPTG). The GST-2×FYVE was purified using the GST-tag Protein Purification Kit (Beyotime) according to the manufacturer’s instructions. Briefly, lysates containing GST-tagged 2×FYVE were incubated with glutathione-Sepharose 4B beads overnight at 4 °C. Then the beads were washed with GST-binding buffer four times, and proteins were eluted, followed by western blotting.

### GST-FYVE labeling

To label PtdIns3P (PI3P) with GST-2×FYVE, cells were fixed and incubated overnight with purified GST-2×FYVE recombinant protein (5 μg/ml) and anti-GST antibody (Origene, 1:1000) at 4°C followed by IF assay.

### Liquid chromatography-tandem–mass spectrometry (LC-MS/MS) analysis

Cells were lysed in lysis buffer (Beyotime) with 1 mM PMSF(Beyotime). Then cellular lysates were incubated with Anti-Cezanne antigen (Proteintech) and protein G Plus-Agarose Immunoprecipitation reagent (Santa Cruz Biotechnology) at 4 °C overnight. After washing and boiling, at least 30 µg samples were subjected to SDS-PAGE. The gel was then stained with Coomassie blue (0.25 g Coomassie blue R250, 45 ml Methanol, 10 ml Acetate, and 45 ml ddH_2_O for 100 ml) and washed with Coomassie Blue Staining Destaining Solution (250 ml Methanol, 80 ml Acetate and 670 ml ddH_2_O for 1000 ml). The stained band was split for LC-MS/MS.

LC-MS/MS was performed by Shanghai Genechem Co., Ltd. (Shanghai, China). The raw data was imported into pFind 3 (Beijing, China) for protein identification. Protein identification was performed using UniProt data. The search parameters included trypsin to generate peptides with a maximum of 3 missed cleavages permitted. A precursor mass tolerance of 20 ppm was specified for MS2 fragments. The carbamidomethyl (C) was set as a fixed modification and variable modifications were Oxidation (M) and Acetyl (Protein N-term). Protein was considered as positively identified if the peptide score of specific peptides reached the significance threshold FDR = 0.01.

### RNA sequencing

The total RNA was extracted using the AG RNAex Pro Reagent (Accurate Biotechnology), and reverse transcription was performed. performed by Annoroad Gene Technology Corporation (Beijing, China). RNA sequencing was performed by Annoroad Gene Technology Corporation (Beijing, China). Briefly, after RNA quality examination, a total amount of 3 μg RNA per sample was used as input material for the RNA sample preparations. Sequencing libraries were generated using NEBNext^®^ Ultra™ RNA Library Prep Kit for Illumina^®^ (NEB, USA) following the manufacturer’s recommendations. RNA concentration of the library was diluted to 1 ng/μl. Insert size was assessed using the Agilent Bioanalyzer 2100 system (Agilent Technologies, CA, USA), and qualified insert size was accurate quantification using StepOnePlus™ Real-Time PCR System (Library valid concentration>10 nM). The clustering of the index-coded samples was performed on a cBot cluster generation system using HiSeq PE Cluster Kit v4-cBot-HS (Illumina) according to the manufacturer’s instructions. After cluster generation, the libraries were sequenced on an Illumina platform and 150 bp paired-end reads were generated.

### Statistical analysis

The TCGA data was analyzed using R software. The differential expression between the two groups was evaluated by the independent Student’s t-test in GraphPad Prism7 (GraphPad Software). Results were shown as mean ± SD. *p* < 0.05 was considered statistically significant. *, **, ***, and **** represent *p* < 0.05, *p* < 0.01, *p* < 0.001, and *p* < 0.0001, respectively.

### Supplementary information

Supplementary Figure Legend

Supplementary Figure 1

Supplementary Figure 2

Supplemental material-WB

Supplemental material-NGS

## Data Availability

Data from TCGA, GSEA, GEPIA, and Cistrome are available in a public, open-access repository. Other analyzing data are available upon reasonable request.
