# Supplementary material for: Cezanne promoted autophagy through PIK3C3 stabilization and PIK3C2A transcription in lung adenocarcinoma
Source: Cell Death Discov. 2023 Aug 18;9:302. doi: 10.1038/s41420-023-01599-4 (PMC10439204; doi:10.1038/s41420-023-01599-4)
Supplement: Supplementary file 1 — Supplementary Figure Legend [file 41420_2023_1599_MOESM1_ESM.docx]

Supplementary Figure

Figure 1. (A)Osimertinib stimulation induced Cezanne expression in PC9 cell. Mean±SD, n = 3. **P < 0.01, vs CT. Osi, Osimertinib. (B) colocalization detection of Cezanne and PIK3C3 in HCC4006 and H1975 cells. Scale bars: 20 µm. (C)Cezanne knockdown did not change the mRNA level of PIK3C3. Mean±SD, n = 3. *P < 0.05, ***P < 0.001, vs siCT. (D) positive correlation between Cezanne and PIK3C3 at protein level. (E) construction of plasmid containing different motifs of Cezanne and the CO-IP assay to identify the binding fragment of Cezanne with PIK3C3. (F) the purified GST-FYVE detected by western blot. Cl, Cell lysates. EL, Elute lysates.

Figure 2. the conserved lysine residue of PIK3C3.
