# Supplementary figures and images for: Cezanne promoted autophagy through PIK3C3 stabilization and PIK3C2A transcription in lung adenocarcinoma
Source: Cell Death Discov. 2023 Aug 18;9:302. doi: 10.1038/s41420-023-01599-4 (PMC10439204; doi:10.1038/s41420-023-01599-4)

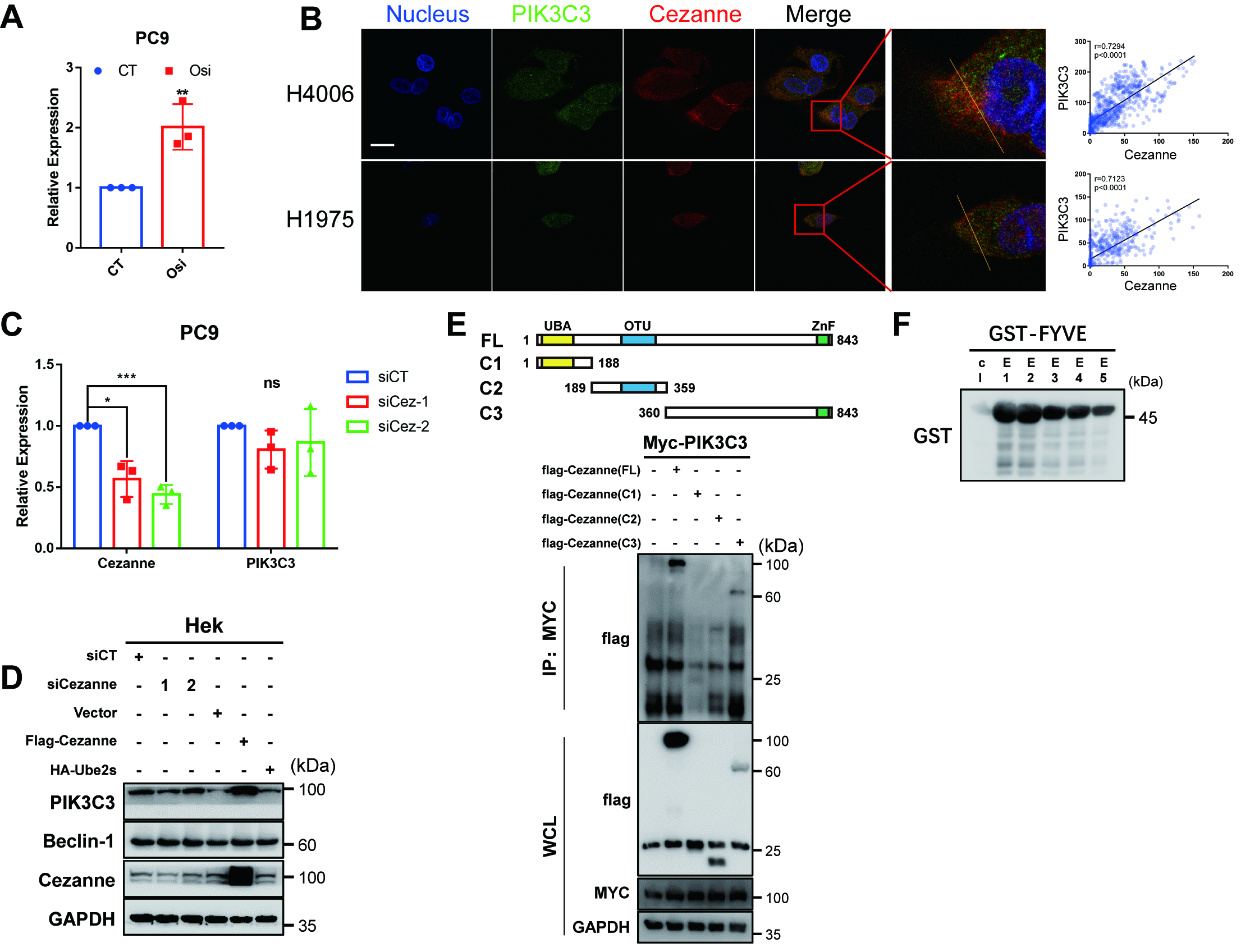

Supplement: Supplementary file 2 — Supplementary Figure 1 [file 41420_2023_1599_MOESM2_ESM.tif]

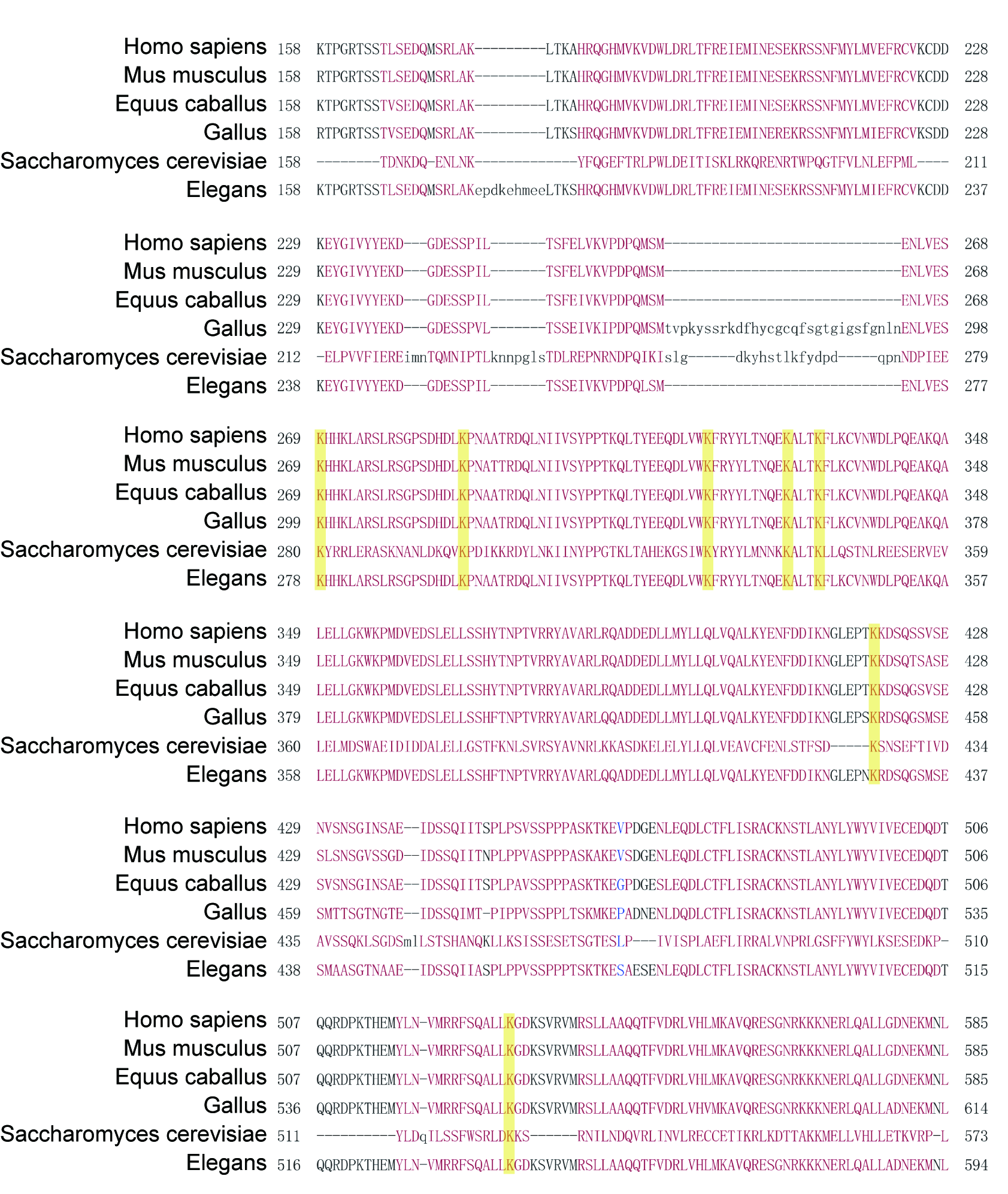

Supplement: Supplementary file 3 — Supplementary Figure 2 [file 41420_2023_1599_MOESM3_ESM.tif]
