# Supplementary material for: Cezanne promoted autophagy through PIK3C3 stabilization and PIK3C2A transcription in lung adenocarcinoma
Source: Cell Death Discov. 2023 Aug 18;9:302. doi: 10.1038/s41420-023-01599-4 (PMC10439204; doi:10.1038/s41420-023-01599-4)

Figure 2B

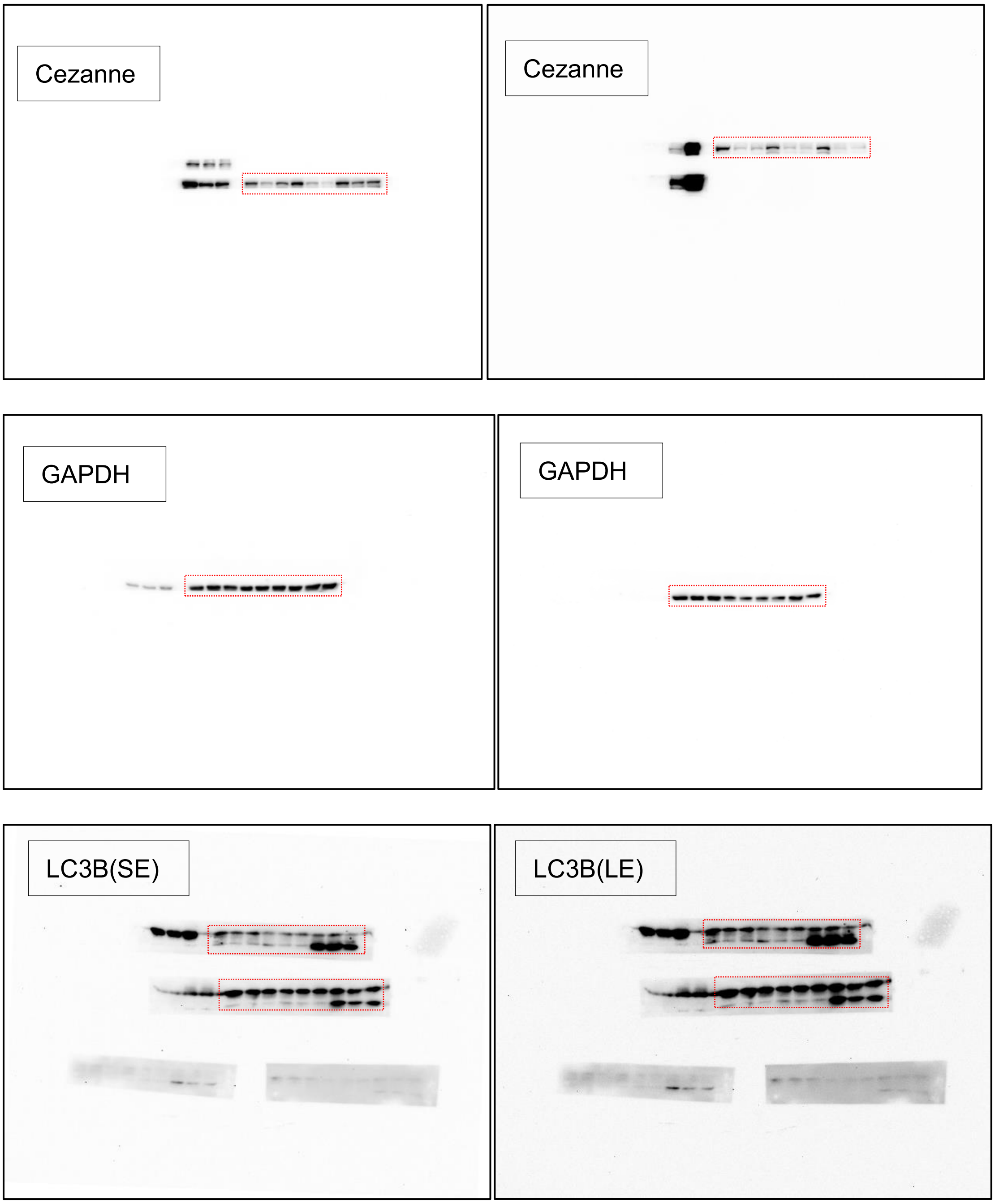


Figure 2C

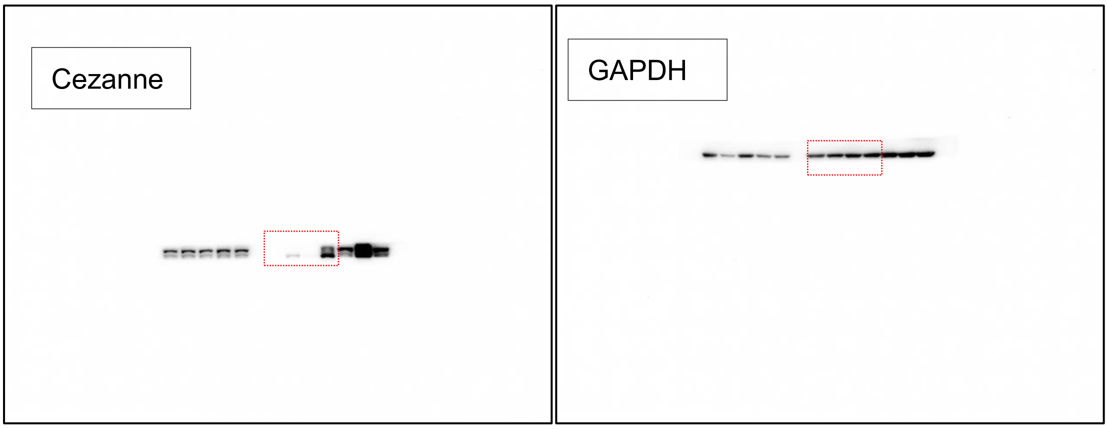

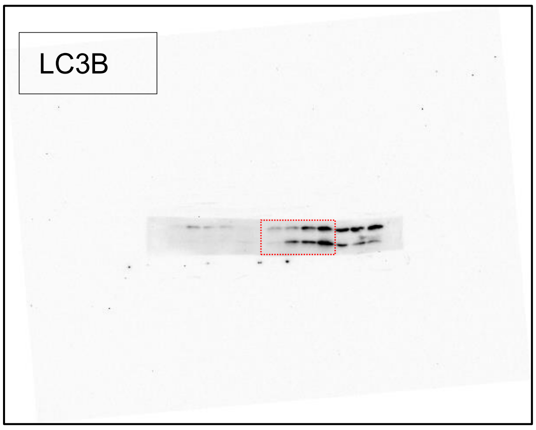


## Figure 2D, F

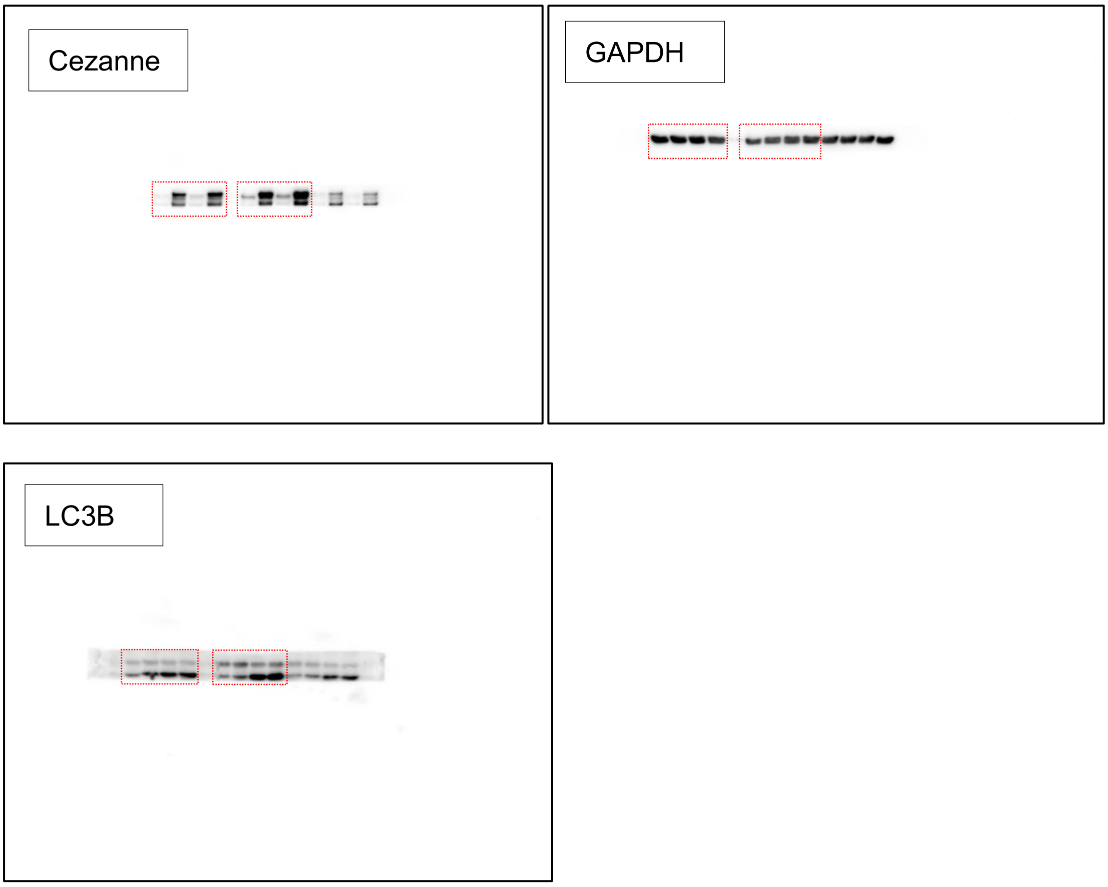


## Figure 2E

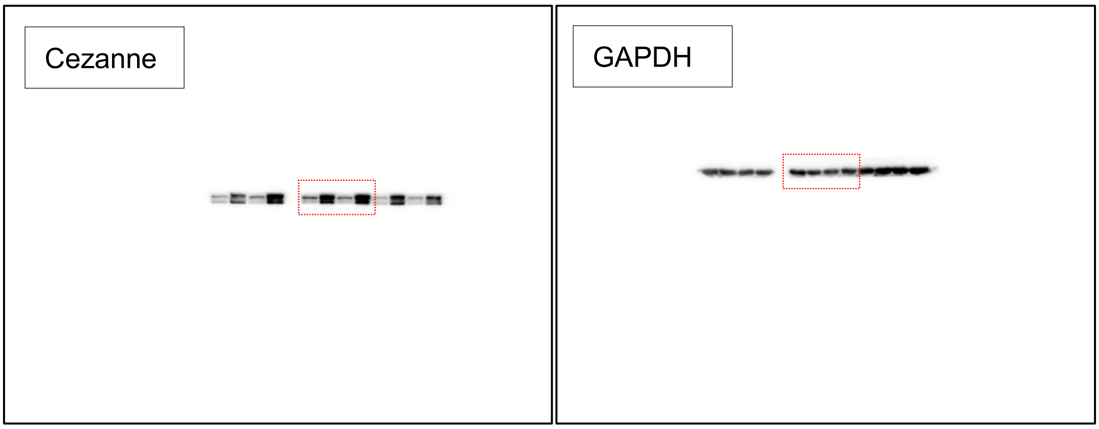

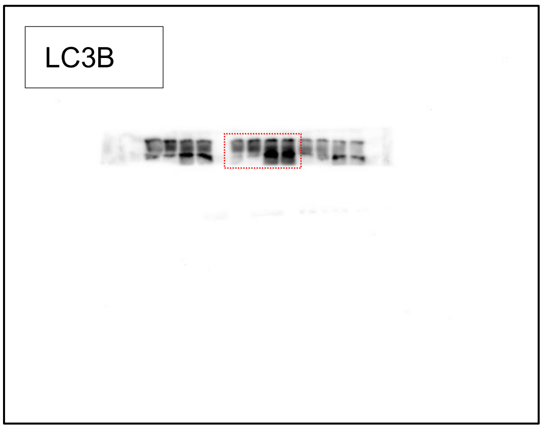


Figure 3B

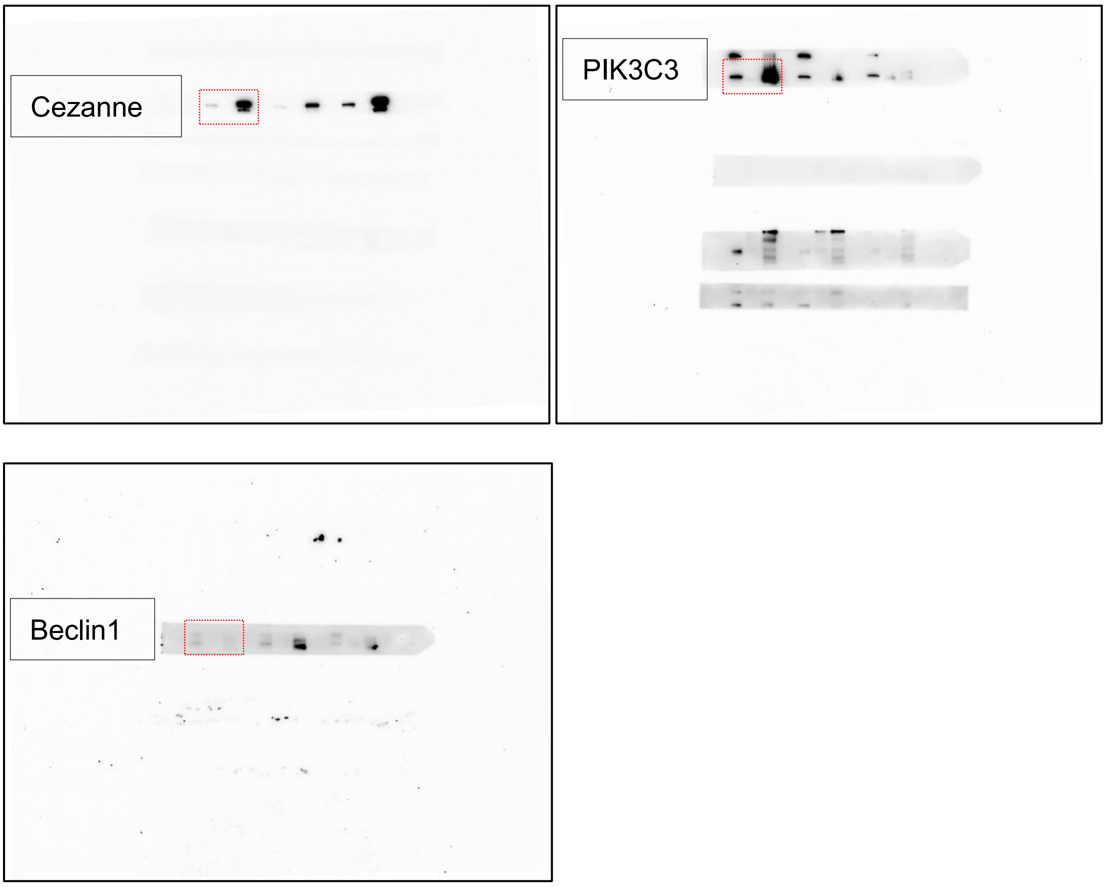


Figure 3C

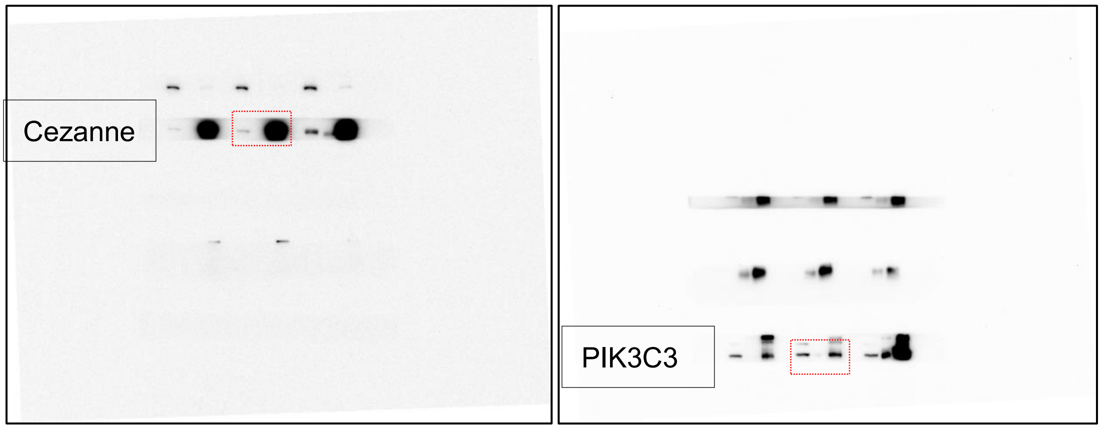

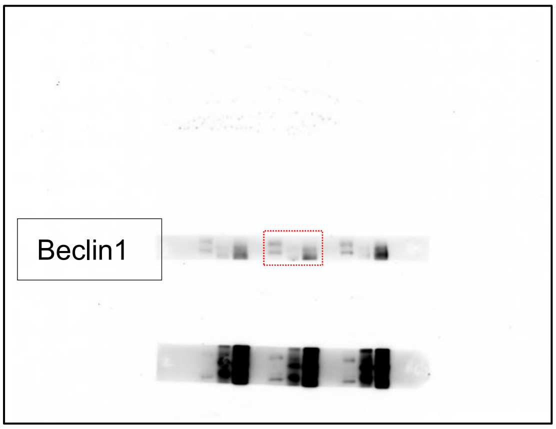


Figure 3D

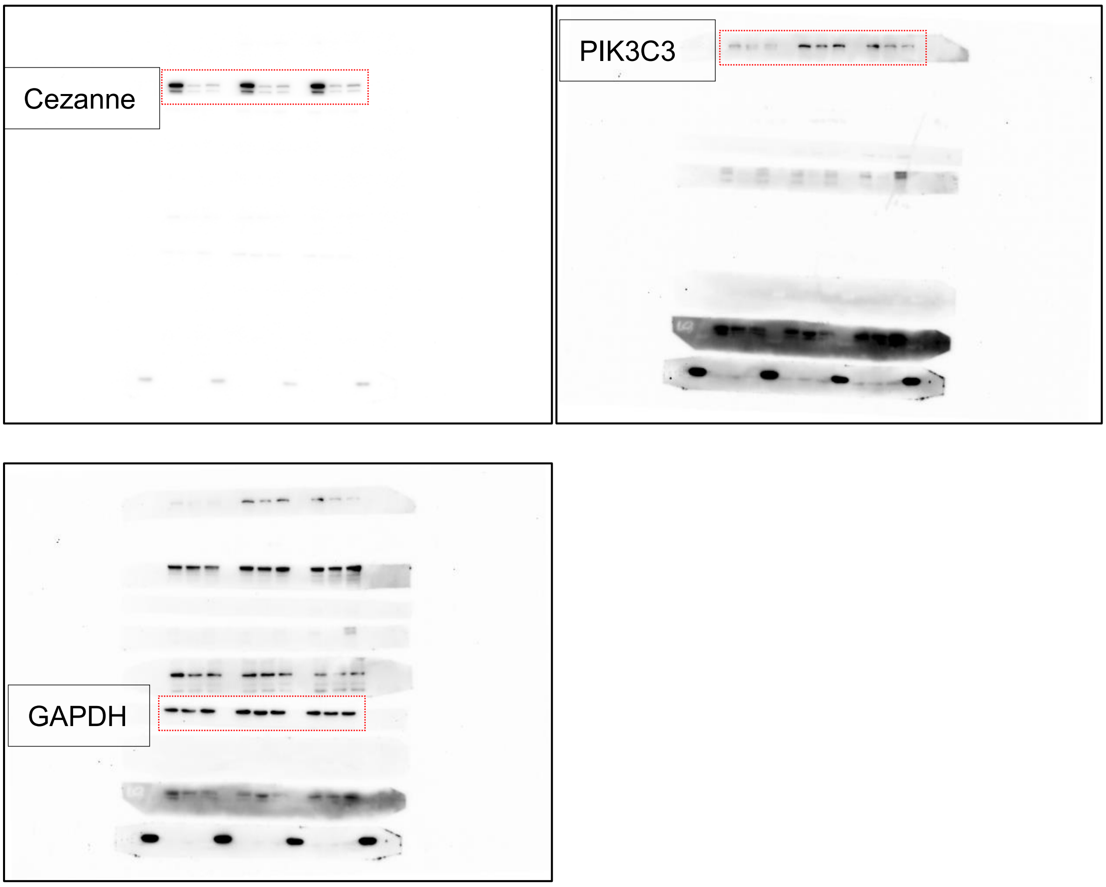


Figure 3E

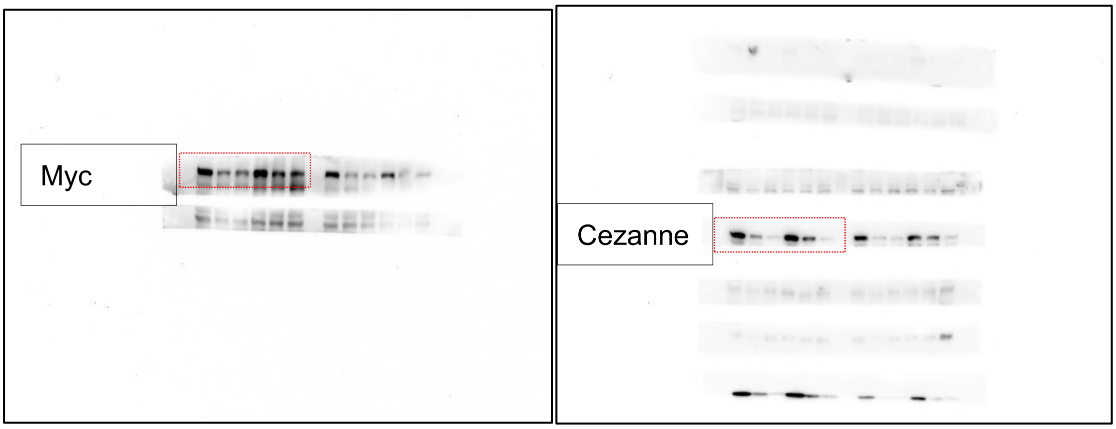

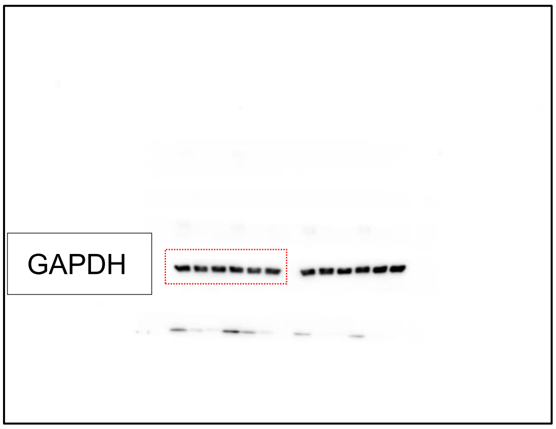


Figure 3F

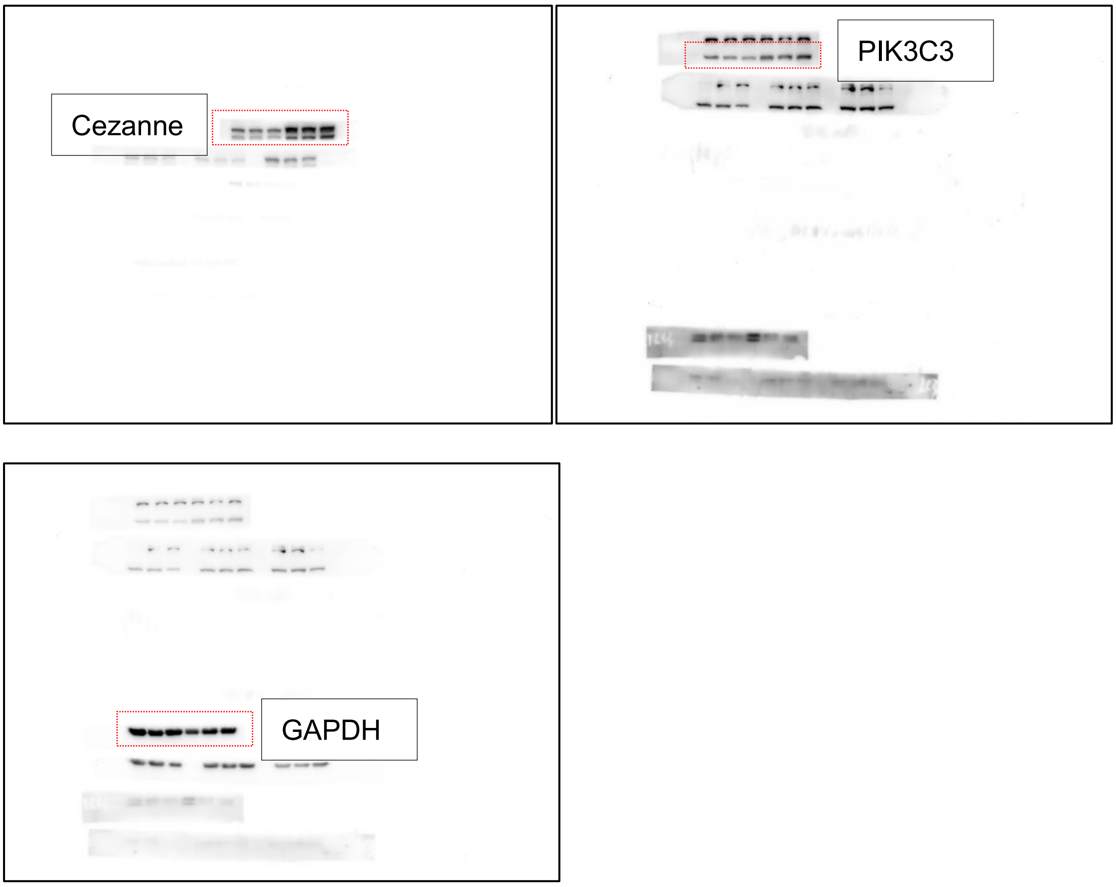


Figure 3G

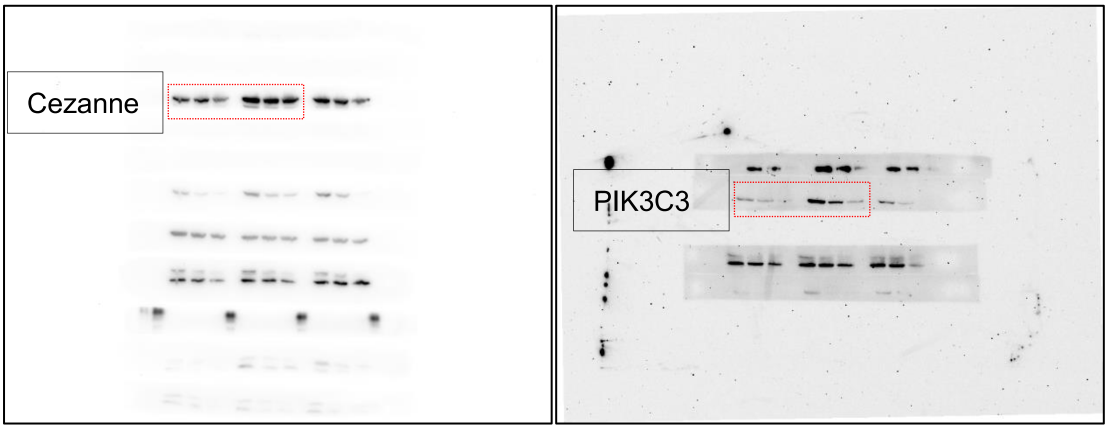

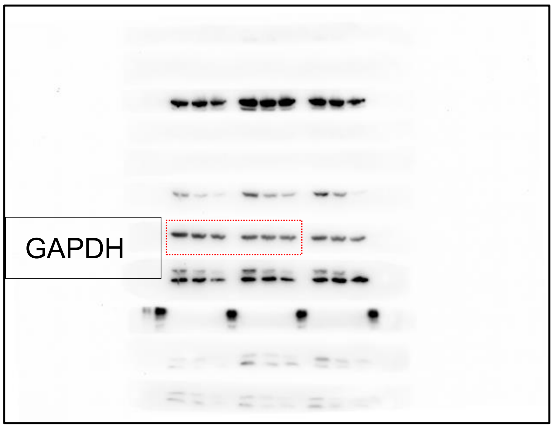


Figure 3H

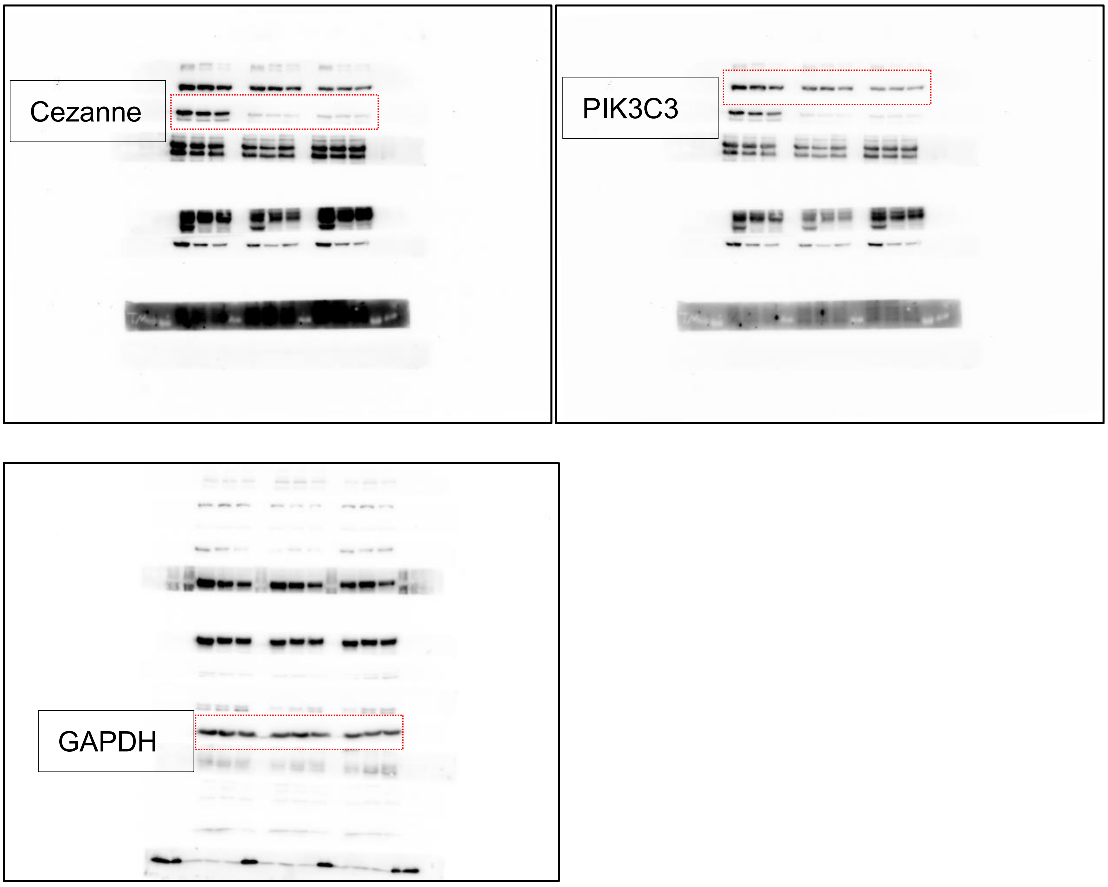


Figure 3I

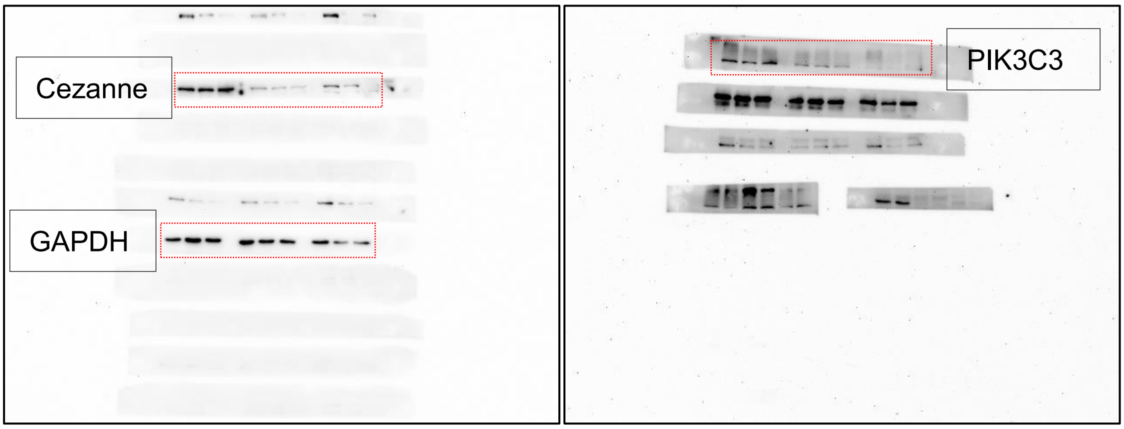

Figure 3J

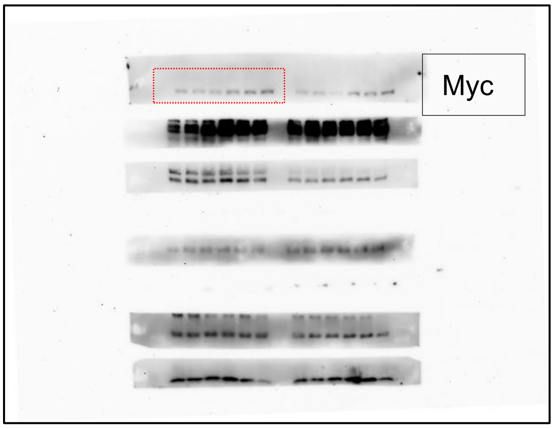


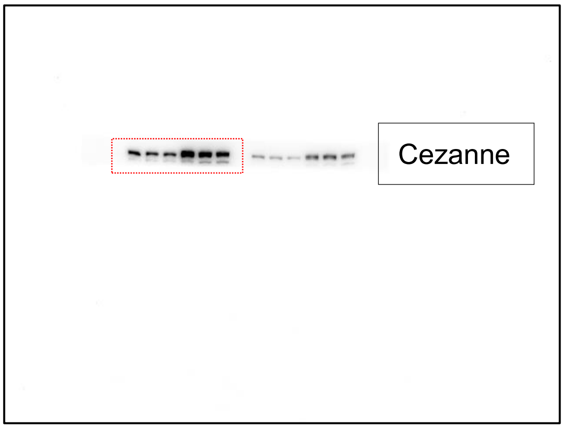


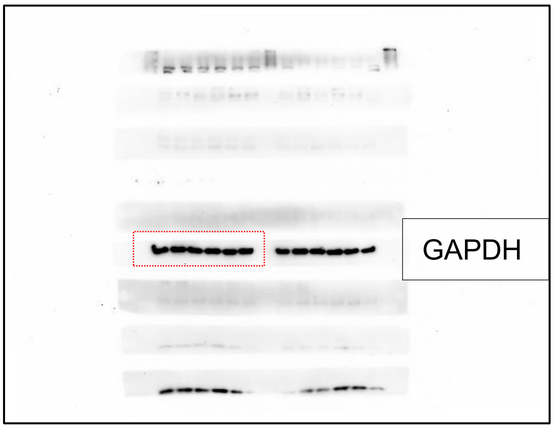


Figure 4A

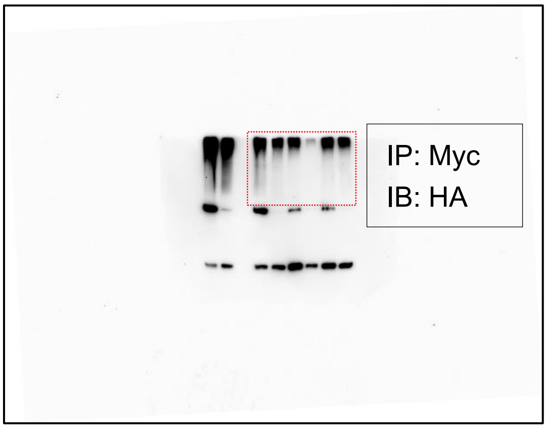


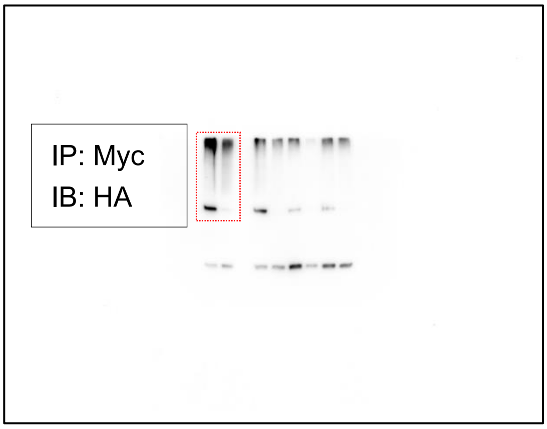


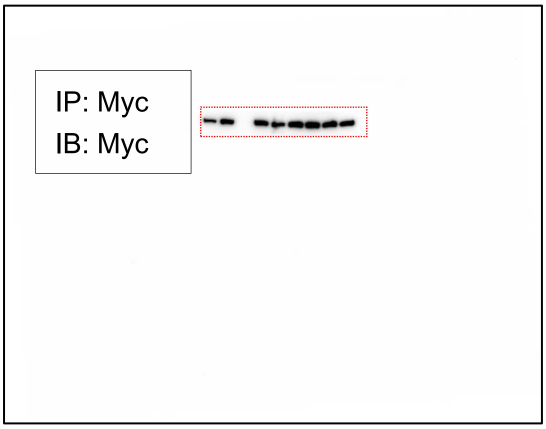


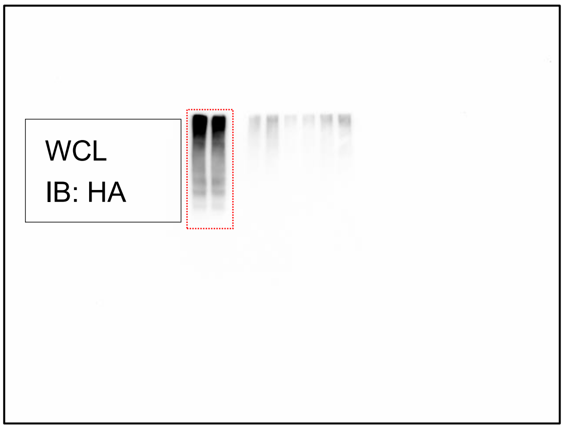

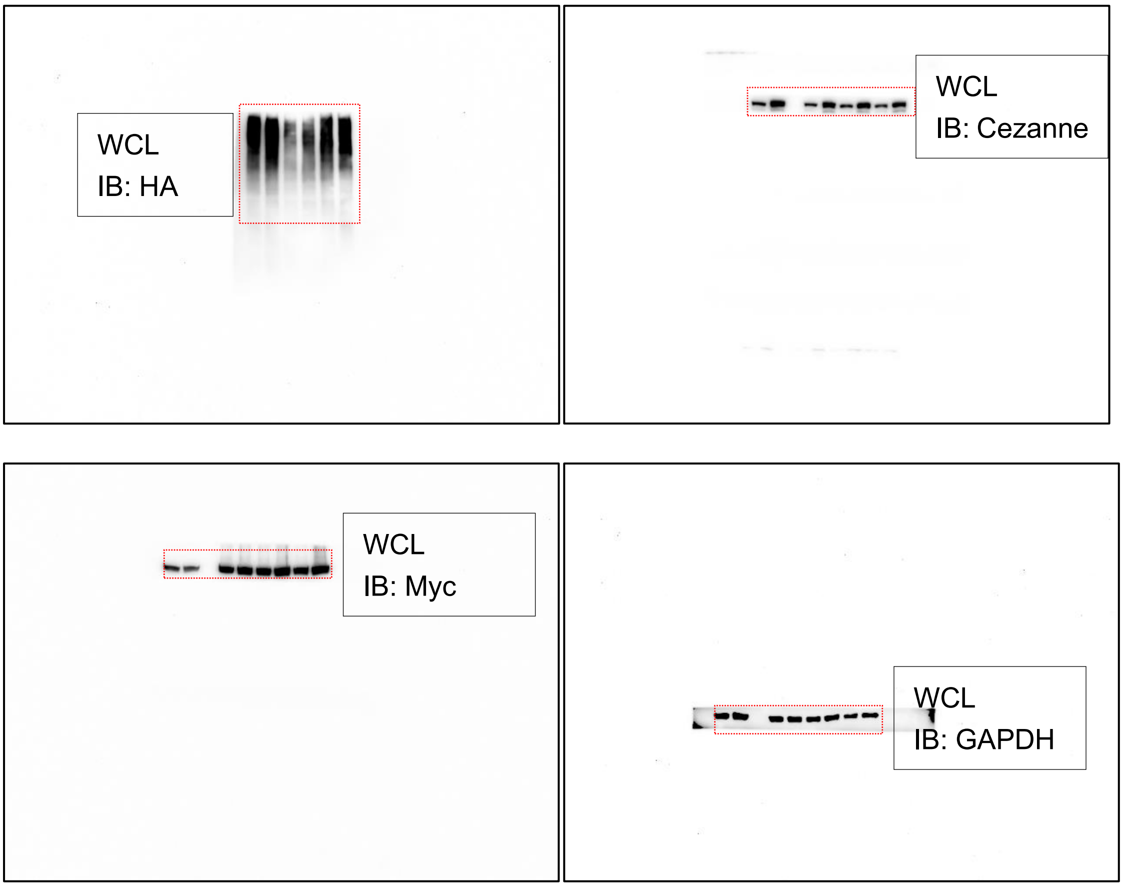
WCL
IB: HA
WCL
IB: Cezanne
WCL
IB: Myc
WCL
IB: GAPDH

Figure 4B

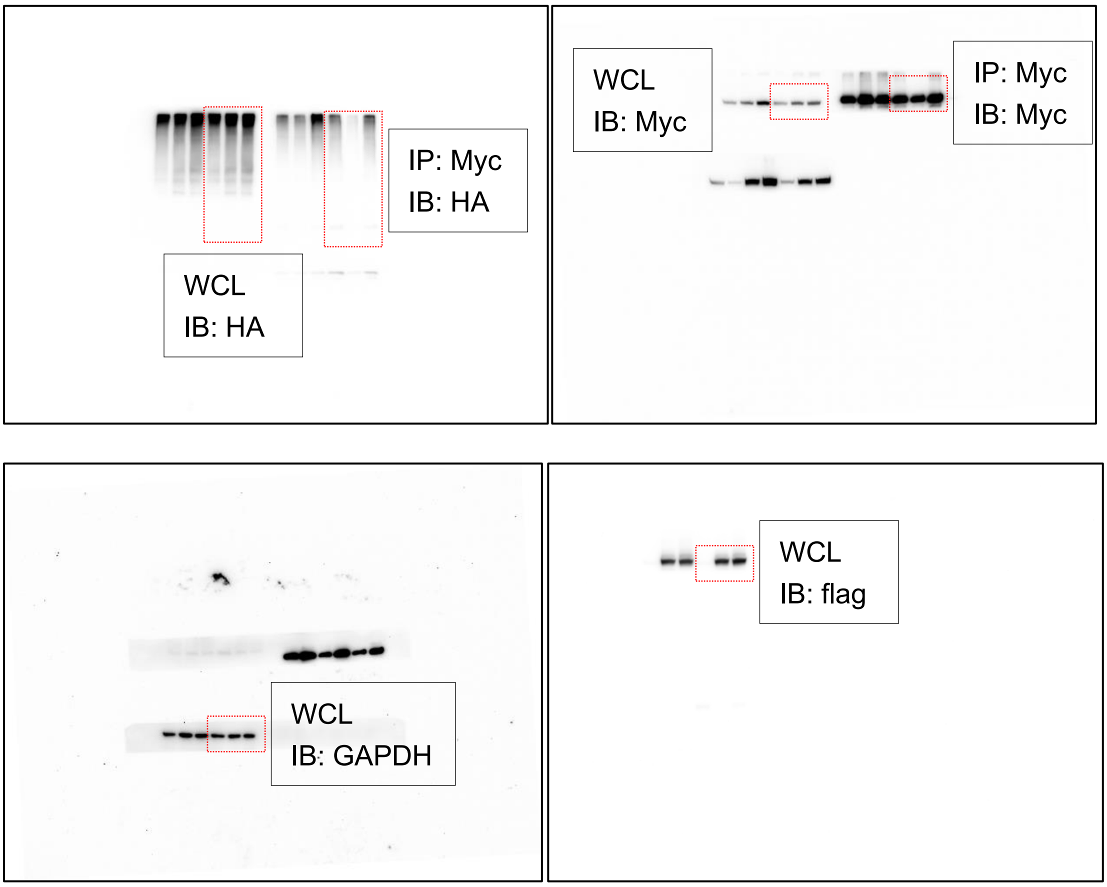
IP: Myc
IB: HA
WCL
IB: HA
WCL
IB: Myc
IP: Myc
IB: Myc
WCL
IB: GAPDH
WCL
IB: flag

Figure 4C

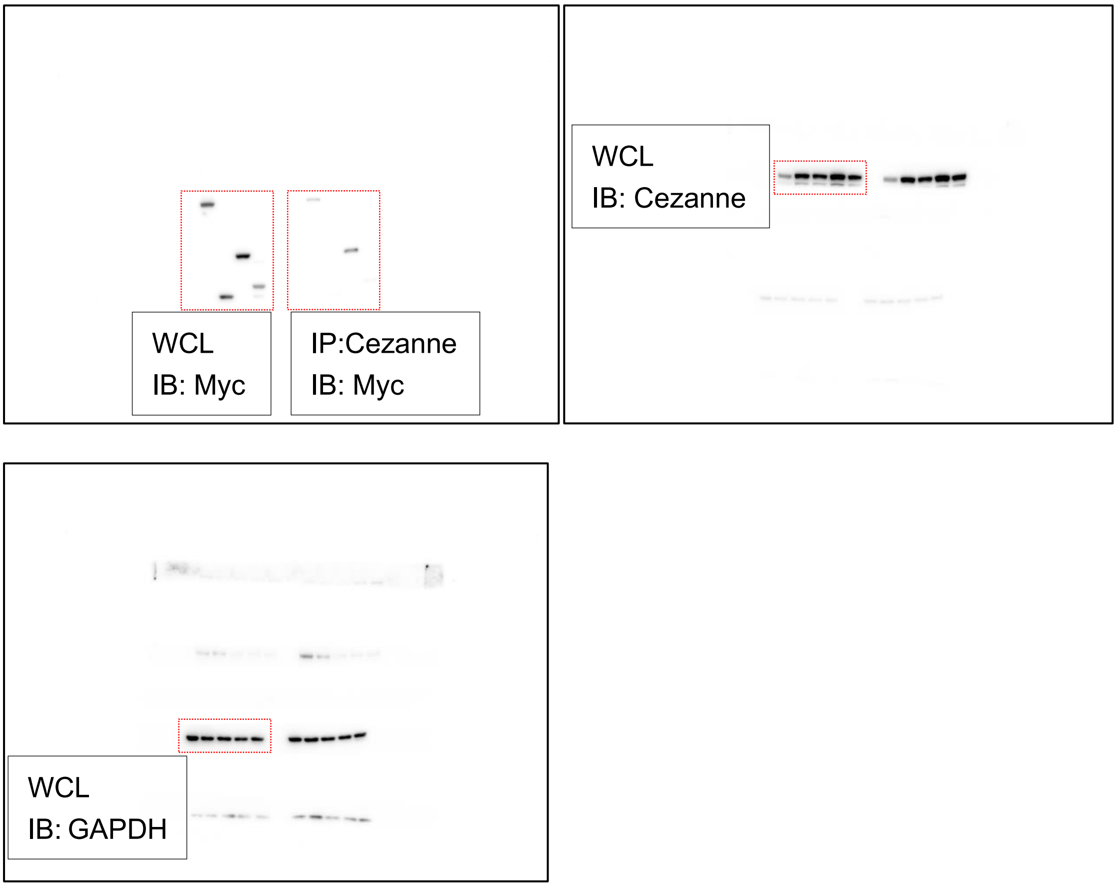


Figure 4D

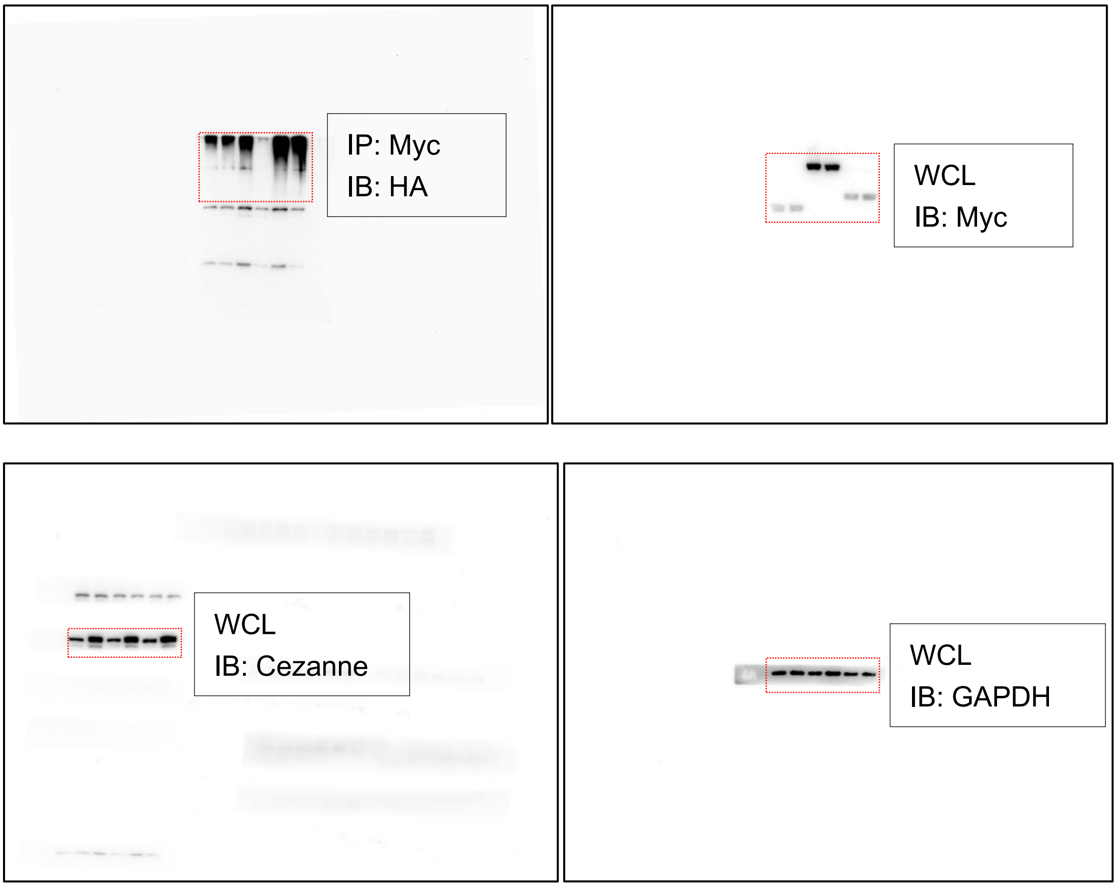

Figure 4E

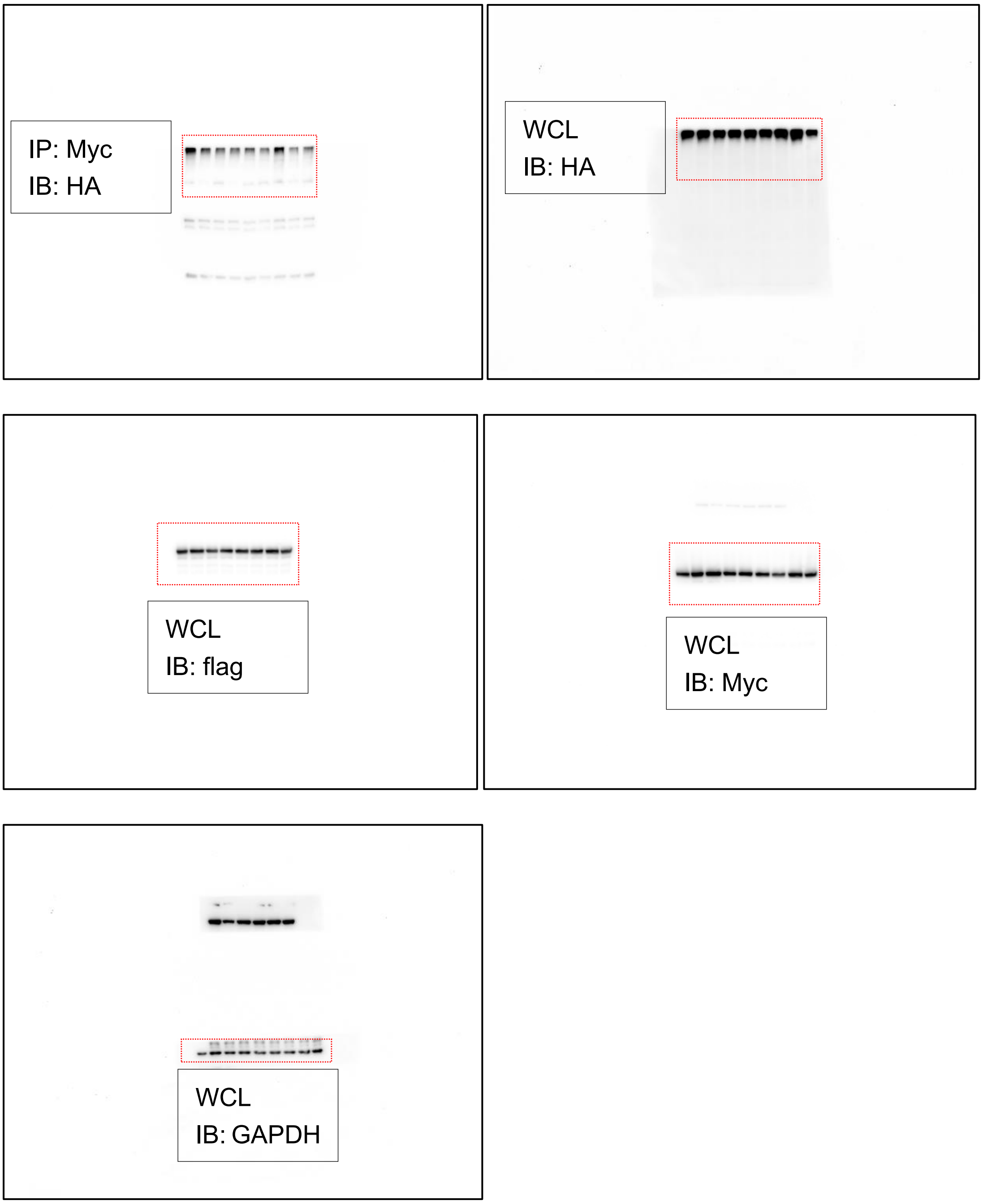


Figure 5D

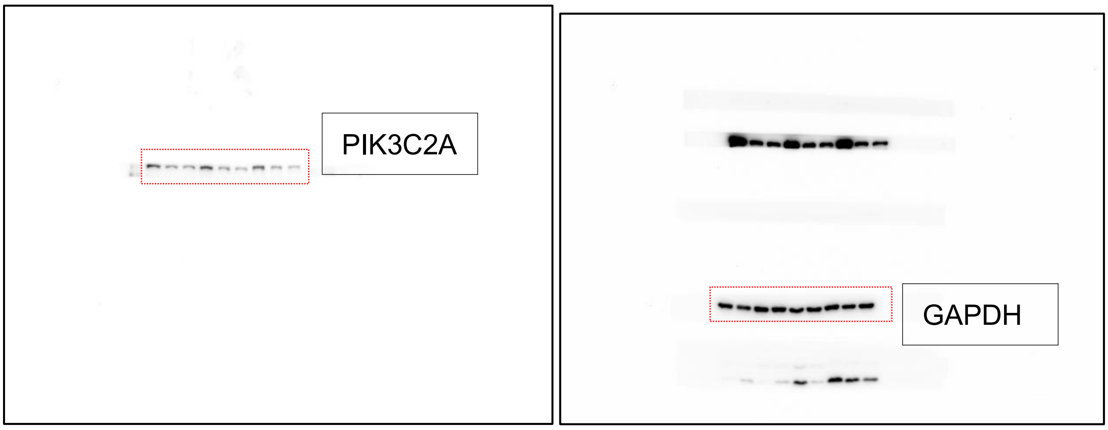

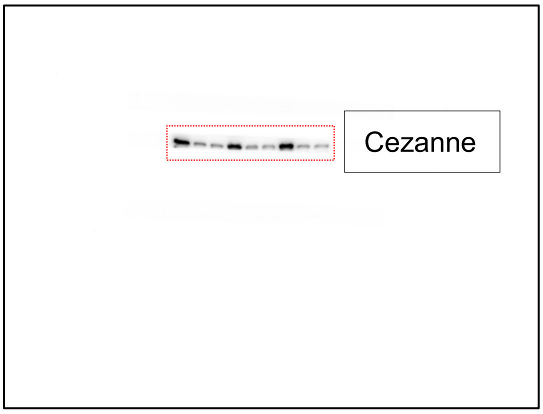


Figure 5L

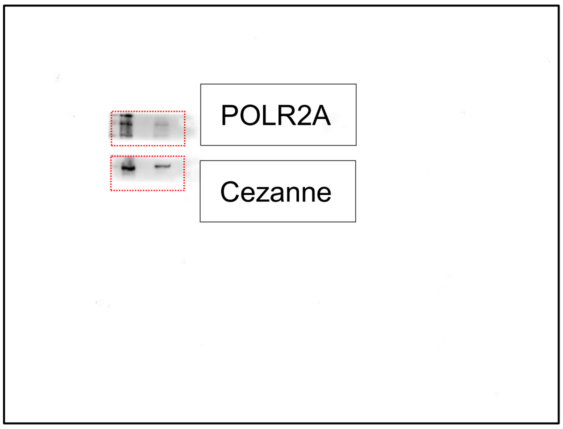


Figure 5M

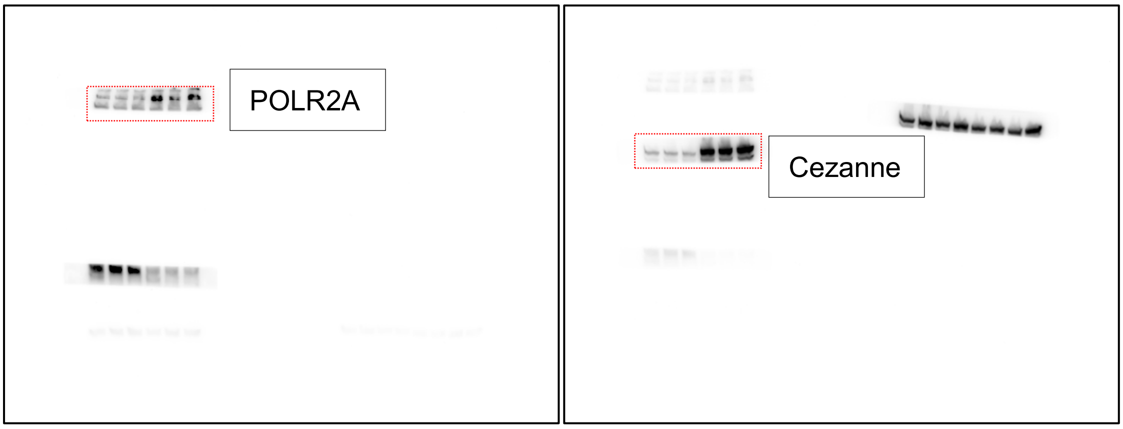


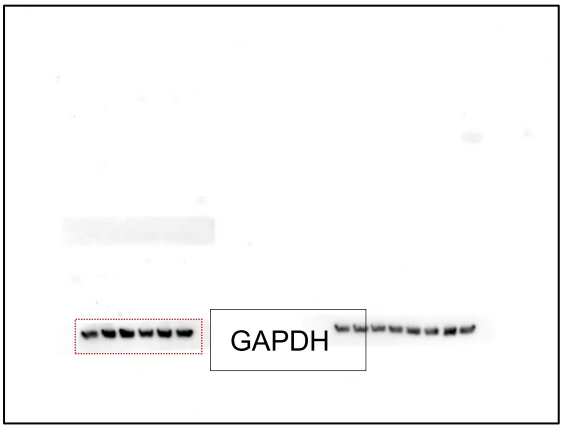


Figure 5N

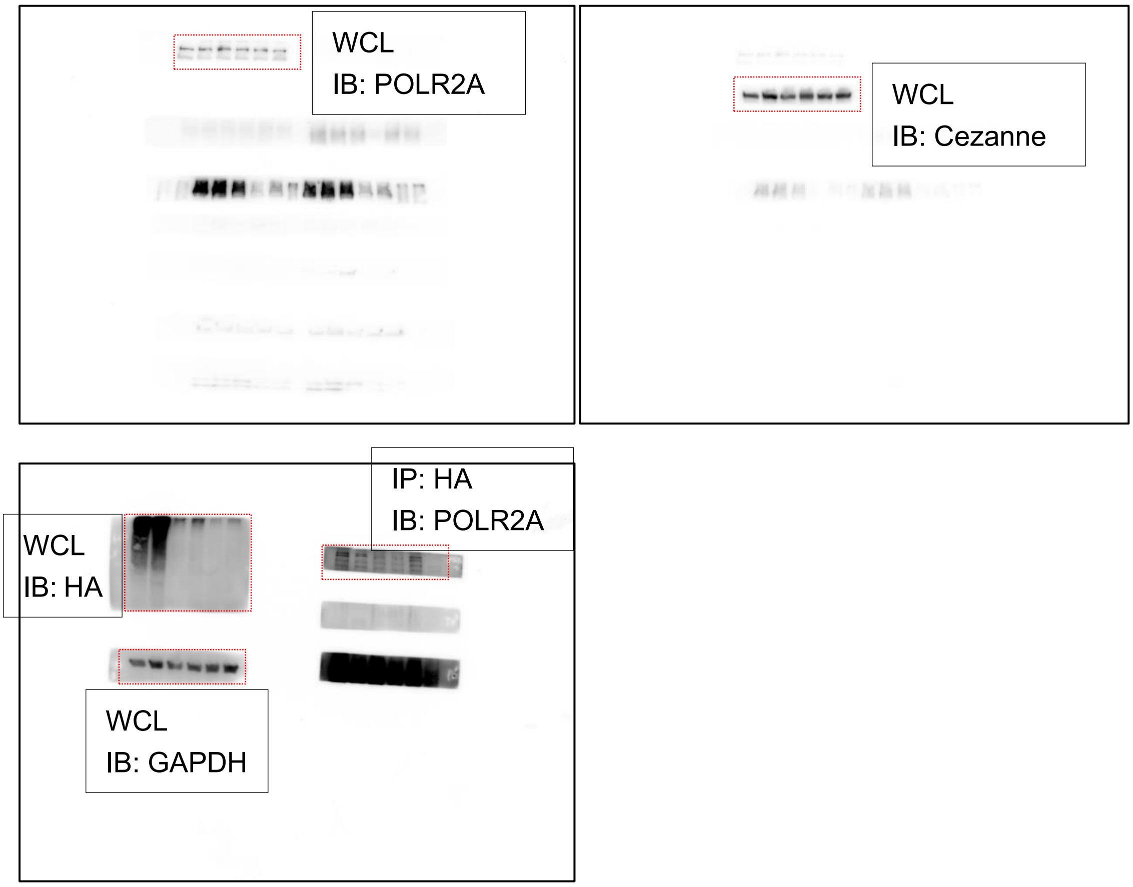


Figure 5O

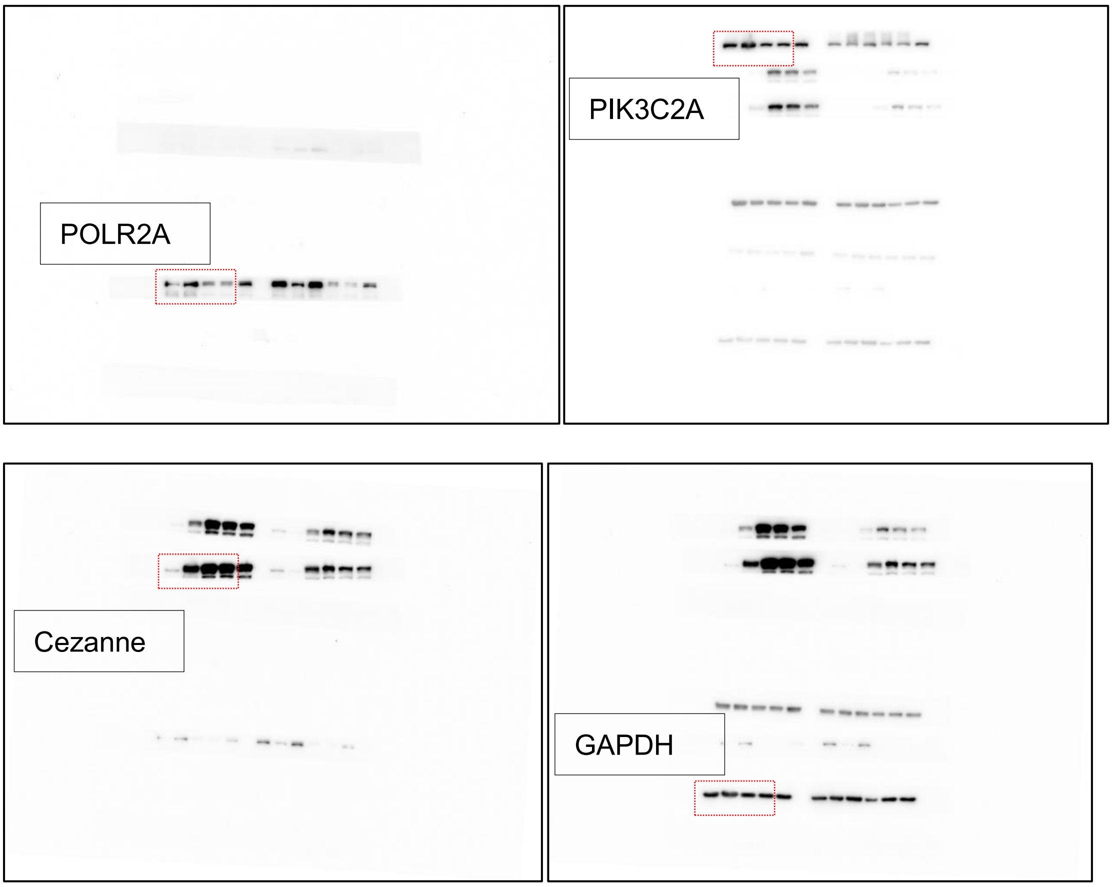


Figure 5P

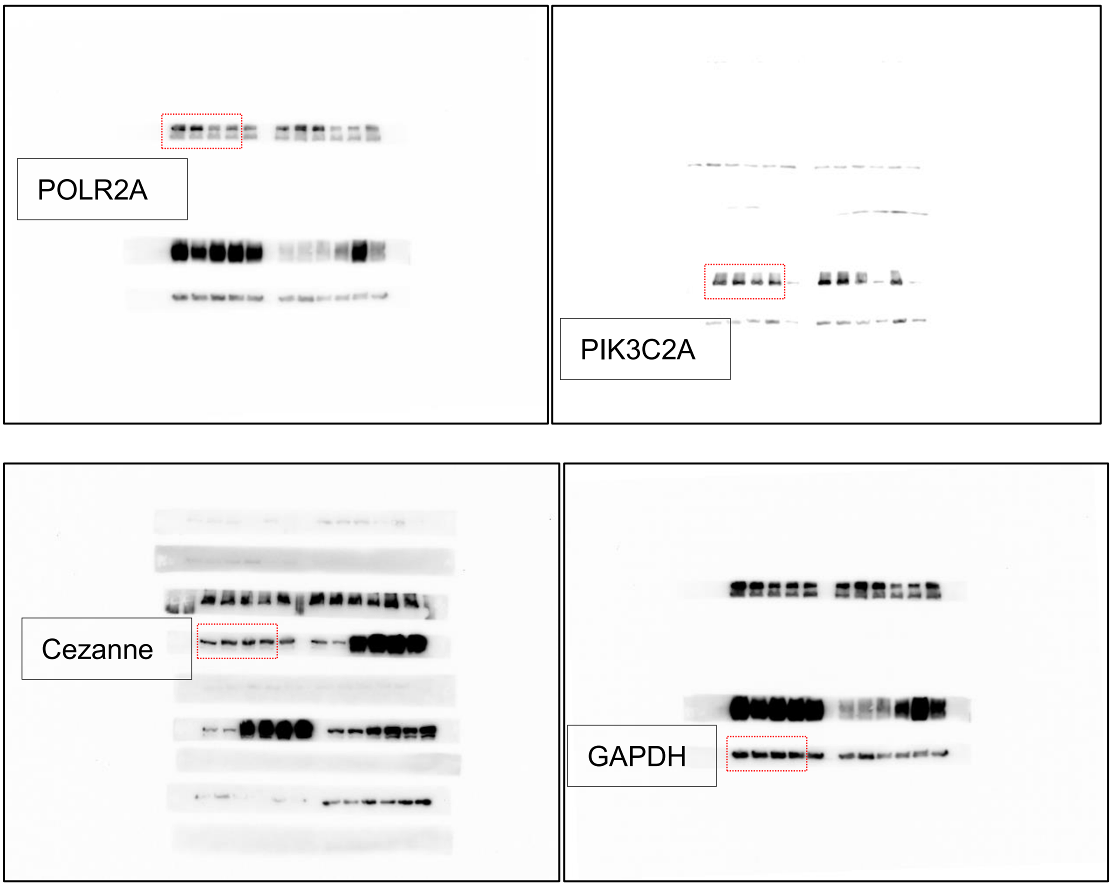


Figure 6E

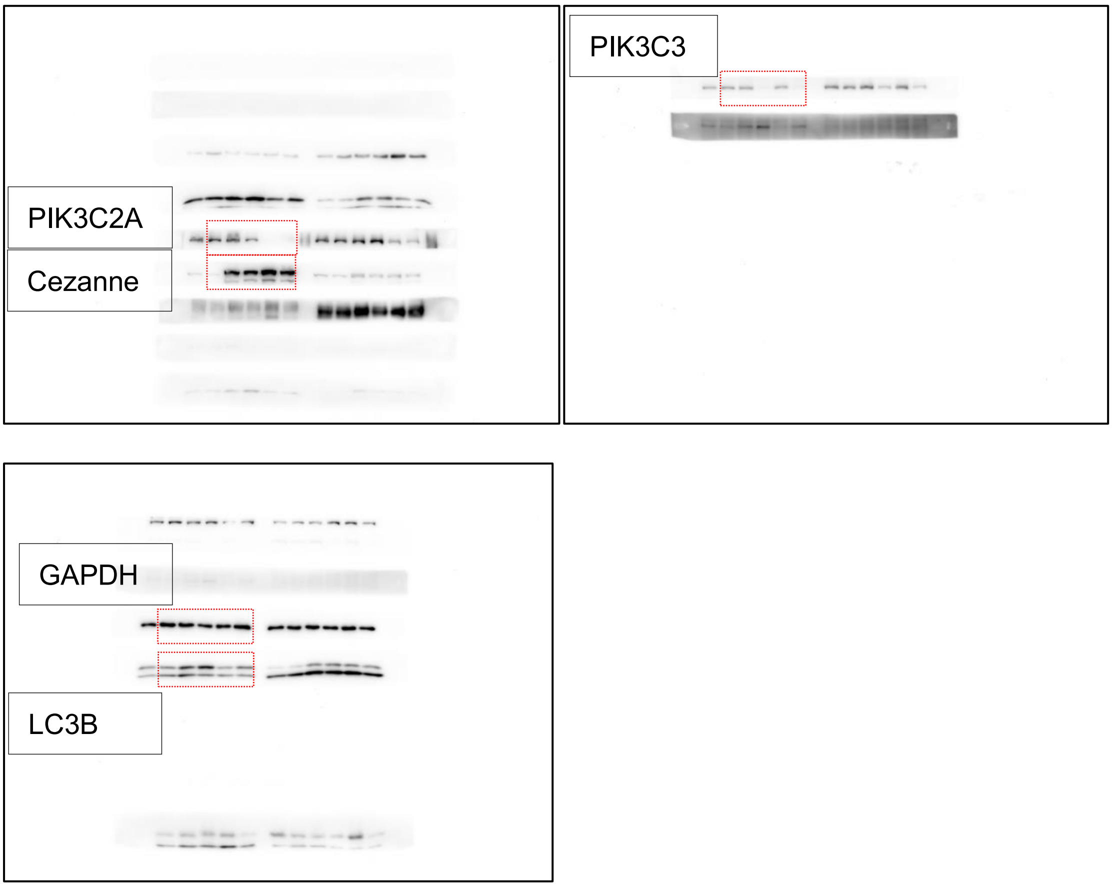


Figure 6F

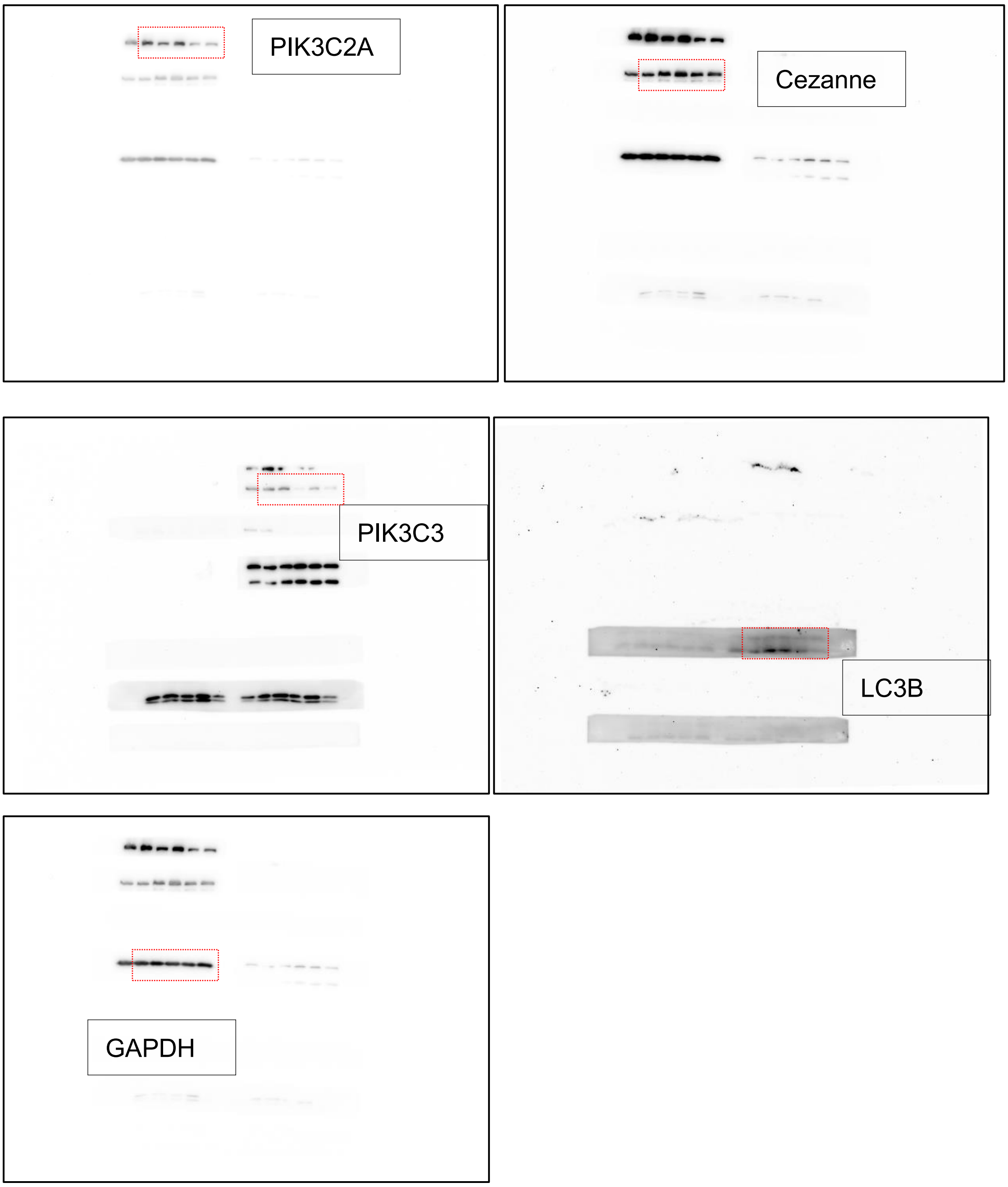


Supplementary Figure 1D

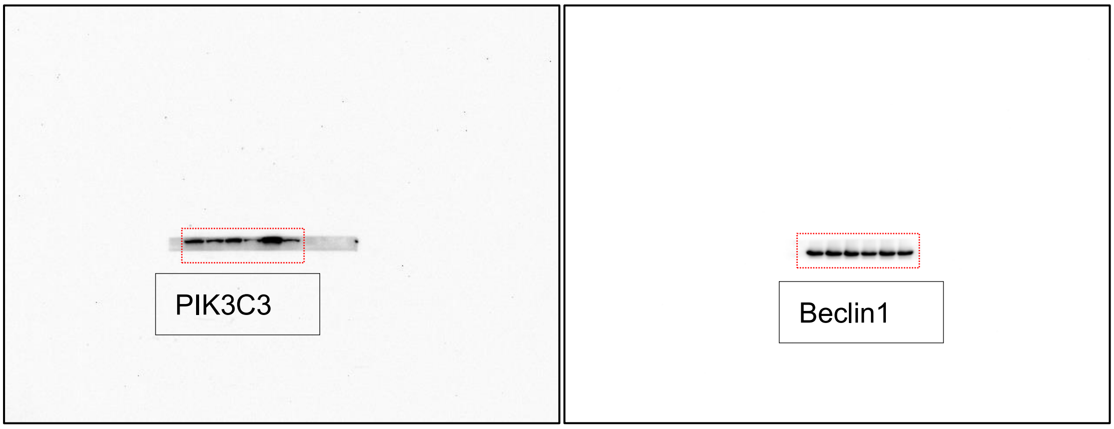

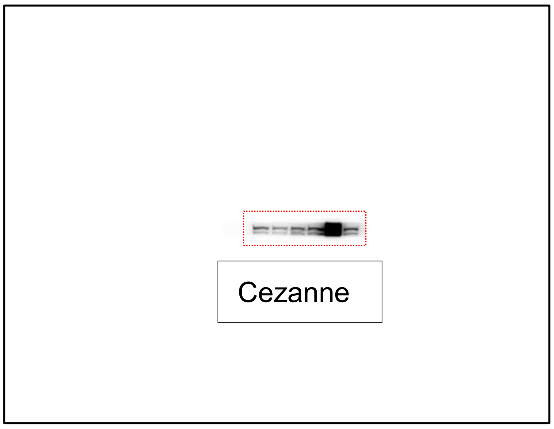


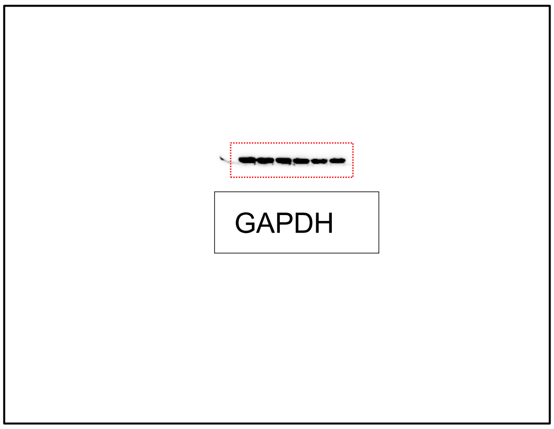


Supplementary Figure 1E

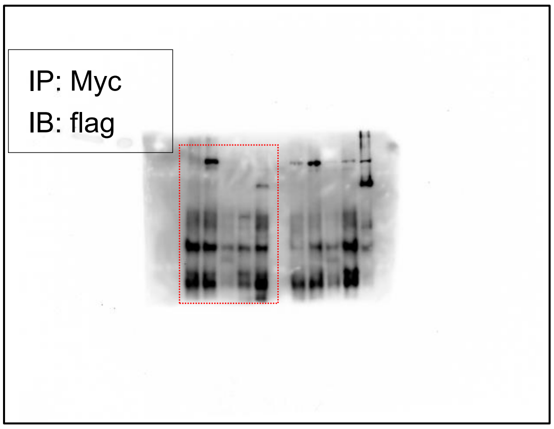


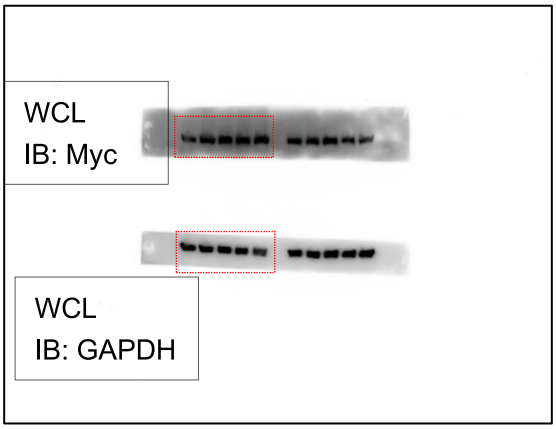


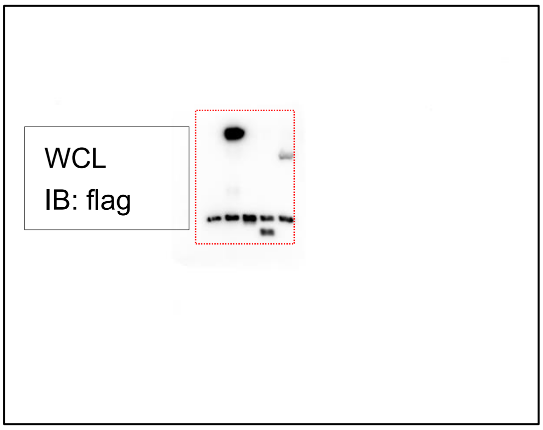


Supplementary Figure 1F

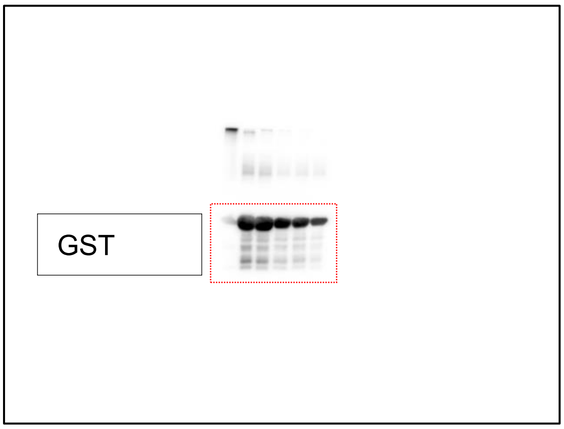

Supplement: Supplementary file 4 — Supplemental material-WB [file 41420_2023_1599_MOESM4_ESM.pdf]
